# Proteomic Profiling of Extracellular Vesicle-Enriched Plasma Using Mag-Net for Biomarker Discovery in Pancreatic Ductal Adenocarcinoma

**DOI:** 10.1021/acs.jproteome.6c00172

**Published:** 2026-06-30

**Authors:** Sindisiwe Buthelezi, Nnenna Elebo, Previn Naicker, Rethabile Mokoena, Sinegugu Dubazana, Ireshyn Govender, Sipho Mamputha, Andrea Ellero, Stoyan Stoychev, Jeanet Mazibuko, Dupe Ojo, Geoffrey Candy, John Devar, Stefano Cacciatore, Jones Omoshoro-Jones, Ekene Emmanuel Nweke

**Affiliations:** † Future Production: Chemicals Cluster, 56417 Council for Scientific and Industrial Research, Pretoria 0001, South Africa; ‡ Department of Surgery, Faculty of Health Sciences, 37708 University of Witwatersrand, Johannesburg 2193, South Africa; § 18470 ReSyn Biosciences, Gauteng 1610, South Africa; ∥ Department of Pharmacology, Faculty of Health Sciences, 56410 University of Pretoria, Pretoria 0007, South Africa; ⊥ 585822 Evosep Biosystems, Billedskærervej 15, Odense 5230, Denmark; # Hepatopancreatobiliary Unit, Department of Surgery, Chris Hani-Baragwanath Academic Hospital, Soweto, Johannesburg, 1860, South Africa; ∇ Department of Integrative Biomedical Sciences, Faculty of Health Sciences, 37716 University of Cape Town, Cape Town 7701, South Africa; ○ Bioinformatics Unit, International Centre for Genetic Engineering and Biotechnology, Observatory, Cape Town 7925, South Africa; ◆ Department of Life and Consumer Sciences, College of Agriculture and Environmental Sciences, University of South Africa, Roodepoort, Florida 1709, South Africa

**Keywords:** pancreatic ductal adenocarcinoma (PDAC), biomarker, plasma, extracellular vesicles, Mag-Net, proteomics

## Abstract

Pancreatic ductal adenocarcinoma (PDAC) is a highly aggressive cancer and was ranked among the top seven leading causes of cancer-related deaths in South Africa in 2020. Highlighting the urgent need for ongoing research to identify reliable biomarkers to improve clinical outcomes. This study compared the plasma proteomes of patients with PDAC, those with benign biliary pathologies (BBP), and healthy controls (HC). We used Mag-Net, a magnetic-bead-based method that enriches membrane-bound vesicles. Comparative analyses identified distinct and overlapping dysregulated proteins between PDAC, BBP, and HC. PDAC showed enrichment for epithelial-mesenchymal transition, complement activation, hypoxia, glycolysis, and extracellular matrix remodeling pathways, consistent with aggressive tumor biology. Proteins including SPP1, THBS2, PTX3, FBLN2, SDC1, CTSS, and VCAN were significantly increased in PDAC, with LRG1 showing the strongest association with disease severity. Proteins, namely GPNMB, H4C1, SAA1, and CCDC47, demonstrated progressive upregulation across disease comparisons, suggesting potential relevance to disease progression. These findings demonstrate that plasma proteomics provides discriminatory molecular insights and supports the development of population-relevant biomarker panels. While several candidate proteins show promise for inclusion in multianalyte panels, further validation in larger cohorts is necessary to establish their diagnostic and translational utility for early detection, risk stratification, and improved differential diagnosis of PDAC.

## Graphical abstract

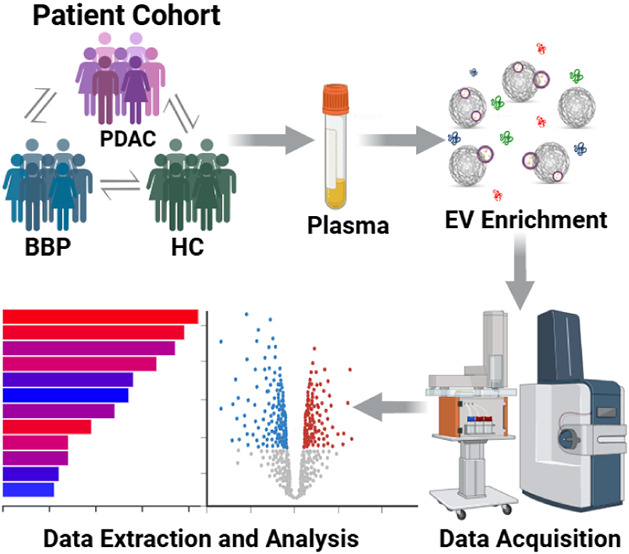

## 1 Introduction

Pancreatic Ductal Adenocarcinoma (PDAC) is an exceptionally aggressive cancer with a 5-year survival rate below 10% and a median survival time of about 6 months.^1,2^ PDAC became the third leading cause of cancer-related mortality worldwide in 2024, with 27,270 and 24,480 estimated mortalities in men and women, respectively.^3^ In South Africa, PDAC is ranked the seventh leading cause of cancer-related deaths in men and sixth in women as of 2020, with over 1900 mortalities.^4^ Although symptoms of PDAC include jaundice, abdominal pain, nausea, vomiting and weight loss, they mostly manifest in advanced stages of the disease.^5^ Hence, the majority of PDAC patients (∼85%) present with the unresectable stage of the disease.^6^ Surgery is the best way to treat PDAC, but this treatment is limited to resectable cases. Other treatment options include chemotherapy, radiation and immunotherapy. Although the mean age at diagnosis of PDAC is around 65 years, recent data suggest that diagnoses of younger individuals with PDAC are on the rise.^7,8^ Environmental and lifestyle factors such as obesity, diabetes and excessive alcohol use have been shown to contribute to the increasing frequency of young-onset PDAC.^7,9^ Other risk factors include genetic factors, chronic pancreatitis, among others.^10^

The poor clinical prognosis of PDAC stems from the absence of effective early diagnostic markers and its continued resistance to available therapies.^11^ Proteomic profiling of biological samples has proven valuable for biomarker discovery in cancer research. Proteins are key regulators of cellular processes,^12,13^ such as signaling, adhesion, metastasis, immune evasion, and drug response, making them promising targets for therapeutic or diagnostic interventions.^14^ Importantly, many proteins are released into biofluids not only as soluble molecules but also packaged within extracellular vesicles (EVs), which can carry and protect protein cargo in circulation. EVs are membrane-bound particles released by nearly all cell types, including tumor cells. They carry a diverse repertoire of biomolecules such as proteins, lipids, and nucleic acids encapsulated within a lipid bilayer that protects the cargo and enables intercellular communication. Through these mechanisms, EVs can influence numerous physiological and pathological processes. This process is connected to several biological functions, including cell communication, angiogenesis, tumor growth, and metastasis.^15,16^ Although recent comprehensive proteomic studies have demonstrated correlations between protein expression and clinical data, a lack of definitive biomarkers for diagnosis and treatment tracking remains a challenge, particularly in African populations.^17,18^ To address this gap, the Mag-Net protocol was used to enrich extracellular vesicles^16^ for analysis of the plasma proteome in a cohort of African patients with PDAC and matched controls, and to compare their expression patterns.

## 2 Materials and Methods

### 2.1 Patient Recruitment and Sample Collection

Ethics approval was obtained by the University of Witwatersrand Human Research Ethics Committee (Medical) (Study number M220735) and the CSIR Ethics Committee (REF: 361/2021). Each participant provided written informed consent. Patient clinical data were collected using REDCap v9.0. The study site was the Hepatopancreatobiliary Unit at Chris Hani Baragwanath Academic Hospital, Soweto, Johannesburg, South Africa. Only individuals who self-reported African ancestry and were older than 18 years were included in this study. Patients with a clinically and histologically confirmed diagnosis of PDAC were recruited. Individuals, including patients with benign biliary pathologies (BBP) and healthy participants (HC), were recruited from the same hospital as a control group. HCs self-confirmed to be in good health and not taking any regular medication were also recruited.

After collection, blood samples were gently mixed by inverting the tube 3 to 5 times and stored upright at 4 °C until centrifugation. Plasma was obtained by centrifuging blood samples collected via venepuncture into EDTA-coated vacutainer tubes (BD Biosciences, Franklin Lakes, NJ, USA) at 1700 g and 4 °C for 10 min, then stored at −80 °C until use.

### 2.2 Plasma Processing

The enrichment of extracellular vesicles from plasma was accomplished using the Mag-Net protocol as described by ref 16 All steps were performed on a Thermo Scientific KingFisher Flex, utilizing eight 96-deep well plates. The protocol consists of two main parts: the first part involves enriching for membrane particles by depleting highly abundant plasma proteins, while the second part involves protein aggregation capture (PAC) and digestion. The plate setup in the KingFisher Flex is illustrated in Figure 1. For the initial capture step, the eight plates were arranged as follows: Position 1:96-well comb; Plate 2: MagReSyn SAX beads (ReSyn Biosciences, Edenvale, South Africa); Plate 3: Bead Equilibration; Plate 4: Membrane Particle Capture; Plates 5–7: Wash; Plate 8: Solubilization/Reduction. Alkylation was subsequently performed offline in Plate 8.

**Figure 1:**
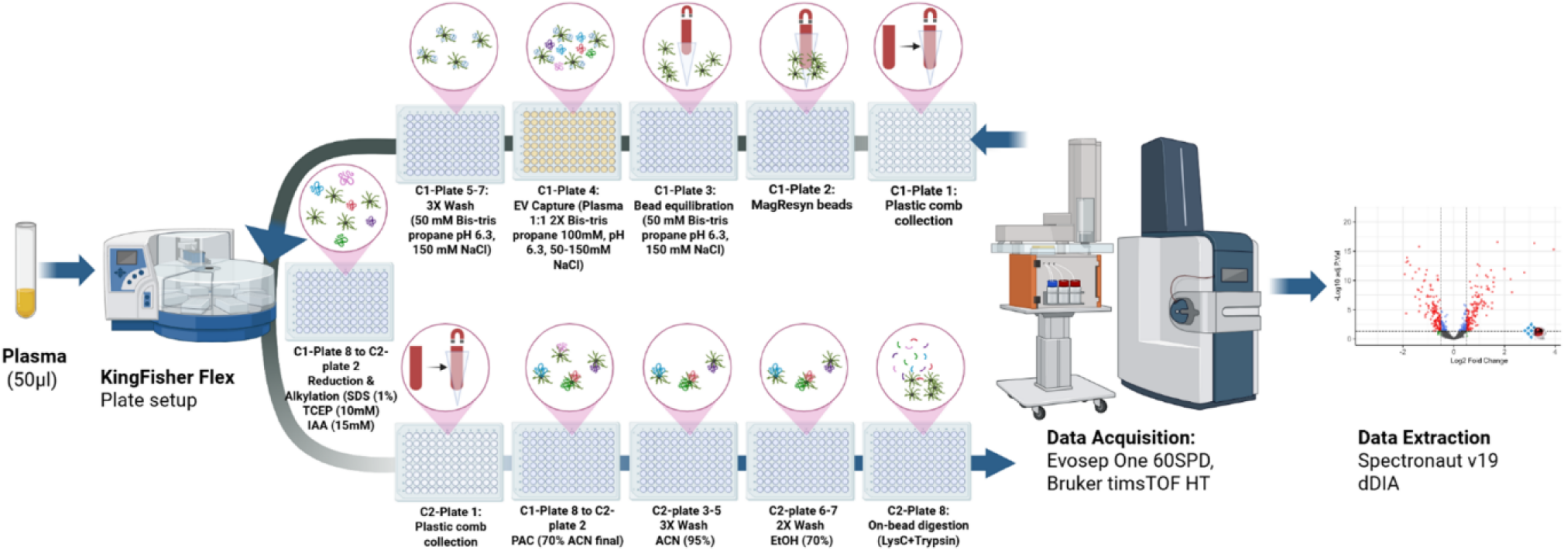
Profiling of the plasma proteome using the Mag-Net protocol. Membrane-bound vesicles were enriched using magnetic hyperporous SAX microparticles (Cycle 1: C1) and then digested on-bead using the PAC (Protein Aggregation Capture) method (Cycle 2: C2). The digested peptides were analyzed using an Evosep One LC system coupled with a timsTOF HT mass spectrometer, and the data were processed in Spectronaut v19 (16).

The second PAC and digestion steps were organized as follows: Position 1:96-well comb; Plate 2: Same Plate 8 from KF part one; Plates 3–5: Acetonitrile Wash; Plates 6–7: Ethanol Wash; Plate 8: Digestion/Peptide Elution with Trypsin (1:20) and LysC (1:200) (Protein: Enzyme ratio). Samples were digested for 2 h at 47 °C. The resulting peptide digests were dried at −4 °C using a vacuum concentrator (CentriVap Concentrator, Labconco, Missouri, USA) and then resuspended in a solution containing 2% acetonitrile and 0.2% formic acid (v/v). The concentration of the peptides was quantified using the Pierce Quantitative Colorimetric Peptide Assay (Thermo Fisher Scientific, Massachusetts, USA), following the manufacturer’s instructions. The samples were then stored at −80 °C until LC-MS/MS analysis.

### 2.3 Liquid Chromatography-Tandem Mass Spectrometry (LC-MS/MS) Data Acquisition

Digested peptides were analyzed using an Evosep One LC system (Evosep Biosystems, Odense, Denmark) coupled with a timsTOF HT mass spectrometer (Bruker Daltonics GmbH & Co. KG, Bremen, Germany). A total of 500 ng of digested peptides per sample were loaded into Evotips according to the manufacturer’s instructions (Evosep Biosystems, Odense, Denmark). The peptides were separated on an 8 cm × 150 μm inner diameter, 1.9 μm C18 analytical column (Evosep ApS, Odense, Denmark), maintained at a temperature of 40 °C, using the standard 60SPD method. The standard dia-PASEF short gradient data acquisition method was employed over a mass range of 100–1000 *m*/*z* and an ion mobility range of 0.85 to 1.30 V·s/cm^2^, utilizing 21 ion mobility windows. The capillary voltage was set to 1600 V, with a ramp and accumulation time of 100 ms. The MS1 and MS2 cycle time was 0.958 s.

### 2.4 Data Processing

The raw data were processed with Spectronaut 19 using the directDIA+ workflow with the default settings applied: specific enzyme cleavage by trypsin/P and LysC/P allowing for 2 consecutive missed cleavage sites, fixed modification was set for cysteine carbamidomethyl, while variable modifications were set for acetyl at protein N-terminal and methionine oxidation. For quantification, no imputation strategy was applied; the protein label-free quantification (LFQ) method applied was automatic, quantity type was set to area under the curve, and quantification was based on MS/MS fragment ion measurements. A search database consisting of all reviewed UniProt protein FASTA sequences, including contaminant proteins from the UniProt Knowledge Database, was used for peptide identification and protein inference. Protein inference was conducted using the IDPicker algorithm.^19^ A log2 transformation was applied to approximate normality, and a median normalization strategy was utilized to evaluate and correct for data biases.

### 2.5 Data and Statistical Analysis

Wilcoxon and Kruskal–Wallis rank-sum tests were used to compare differences in numerical covariates (e.g., age). Fisher’s exact test was used to assess differences between categorical variables (e.g., gender), and Spearman’s rank test was then used to calculate the correlation coefficient (rho) between variables. The threshold for a significant fold change was set to ≥2. This decision was guided by a power analysis conducted in R Studio, which utilized the observed variance in protein abundance between groups. The analysis estimated the minimum detectable effect size with a power of 0.8 and a q-value of 0.05. Receiver operating characteristic (ROC) analysis was used to evaluate discrimination between control groups and PDAC. The area under the ROC curve (AUC) was computed with 95% confidence intervals (95% CI) estimated by DeLong’s nonparametric method.

Performance was assessed through exploratory data analysis tools such as Principal Component Analysis (PCA). Protein identifiers were mapped to corresponding gene symbols using the appropriate annotation database. Gene set enrichment analysis was conducted on Enrichr (20–22) and further examined with the FGSEA algorithm,^23^ applying the Hallmark gene sets from the Molecular Signatures Database (24) (MSigDB 2020) and to evaluate the overrepresentation of tumor-associated pathways, in combination with pathway annotations from the KEGG (2021) database.^24,25^ The correlation map was built by applying the KODAMA algorithm^26–28^ to the first 20 dimensions of the multidimensional scaling computed on Spearman’s correlation distance matrix. A shared nearest neighbor graph was constructed from the KODAMA projection using k = 10 nearest neighbors. Protein cluster detection was then performed with the Walktrap algorithm.^29^

## 3 Results

### 3.1 Demographic and Clinicopathological Characteristics of PDAC Patients

A total of 81 PDAC patients and 97 controls, including 62 BBP and 35 HC, were recruited. BBP cases were predominantly female (85.5%), whereas PDAC was more frequent in males (67.1%; p-value < 0.01). In contrast, the gender distribution among healthy controls was more balanced (42.9% female vs 57.1% male). Age differed significantly across groups: the median age was 31 years in HC, 39 years in BBP, and 62 years in PDAC (p-value < 0.01). The clinical features were compared across 62 patients with BBP and the three stages of PDAC, including 60 with resectable pancreatic cancer (RPC), 15 with locally advanced pancreatic cancer (LAPC), and 6 with metastatic pancreatic cancer (MPC), as reported in Table 1. C-reactive protein (CRP) increased significantly with disease severity, rising progressively from BBP through RPC and LAPC to MPC. Significantly elevated alkaline phosphatase (ALP) and gamma-glutamyl transferase (GGT) levels were observed in PDAC when compared to BBP. The abnormal bilirubin values observed in PDAC are an expected consequence of cholestatic jaundice, a condition commonly associated with the disease.

**Table 1:** Comparison of Clinical Parameters of Biliary Benign Pathologies and Pancreatic Ductal Adenocarcinoma^a^

Feature	N	BBP Median [IQR]	RPC Median [IQR]	LAPC Median [IQR]	MPC Median [IQR]	p-Value
WCC	121	8.14 [6.7 10.3]	9.2 [7.2 13.1]	9.7 [6.6 11.3]	14.0 [6.5 17.0]	0.598
Haemaglobin	122	12.0 [10.5 13.6]	10.2 [9.0 11.6]	10.0 [8.1 11.8]	11.4 [10.9 12.7]	<0.01
Creatinine (umol/L)	120	71 [61 88]	75 [58 98]	68 [57 81]	75 [66 78]	0.837
CRP (mg/L)	121	26 [9 105]	65 [19 149]	94 [39 136]	134 [41 288]	0.03
Total Protein (g/L)	104	72 [68 78]	64 [57 68]	66 [56 67]	71.5 [69 75]	<0.01
Albumin (g/L)	113	39 [35 42]	31 [28 35]	27 [25 31]	36 [26 39]	<0.01
Tbil (umol/L)	130	27 [8 71]	154 [81 264]	90 [29 179]	56 [40 60]	<0.01
Dbil (umol/L)	130	17 [3 64]	141 [63 238]	79.5 [18.25 147]	39 [38 51]	<0.01
ALT (U/L)	113	36 [15 89]	98 [48 151]	61 [23 94]	46 [30 48]	0.02
AST (U/L)	113	47 [25 72]	127 [68 162]	96 [53 117]	108 [42 123]	<0.01
ALP (U/L)	130	177 [106 329]	634 [305 1159]	337.5 [182 733]	327 [302 1645]	<0.01
GGT (U/L)	130	144 [73 362]	725 [307 1175]	301 [146 638]	566 [400 938]	<0.01

a Abbreviations: BBP, Benign biliary pathologies; RPC, Resectable pancreatic ductal adenocarcinoma; LAPC, Locally advanced pancreatic ductal adenocarcinoma; MPC, Metastatic pancreatic ductal adenocarcinoma; WCC, White cell count; CRP, C-reactive protein; Tbil, Total bilirubin; Dbil, Conjugated bilirubin; ALT, Alanine transaminase; AST, Aspartate transaminase; GGT, Gamma glutamyl transferase; N, Number of patients.

### 3.2 Characterization of the Plasma Proteome

On average, 2,339 protein groups were identified across all sample groups using the Mag-Net approach. PCA of the normalized and filtered data revealed that the samples cluster reasonably well by clinical conditions (PDAC, BBP, and HC); however, there is greater heterogeneity within the PDAC and HC groups along PC2, which may reflect underlying intragroup variation. The overlap between BBP and PDAC, while the HC group is almost spatially resolved from PDAC, is also interesting and aligns with the clinical classifications (Figure 2). In the second and third principal components (PC2 and PC3), the BBP samples occupy an intermediate position, overlapping with both the PDAC and HC groups. The PC1 versus PC2 projection primarily captured sources of variance that did not result in clear spatial resolution of the biological group, as illustrated in Supporting Information: PCA of proteomics data showing the PC1 versus PC2 projection and assessment of sample variance distribution across biological cohorts (Figure S9). Supporting Information: LC–MS quality-control assessment, including HeLa protein digest (HeLa QC) and pooled-sample peptide quality-control (Pool QC) analyses showing protein group and peptide identification reproducibility, replicate correlation analysis, QC variability assessment, and LC peak performance parameters (Figures S1–S7 and Table S4).

**Figure 2:**
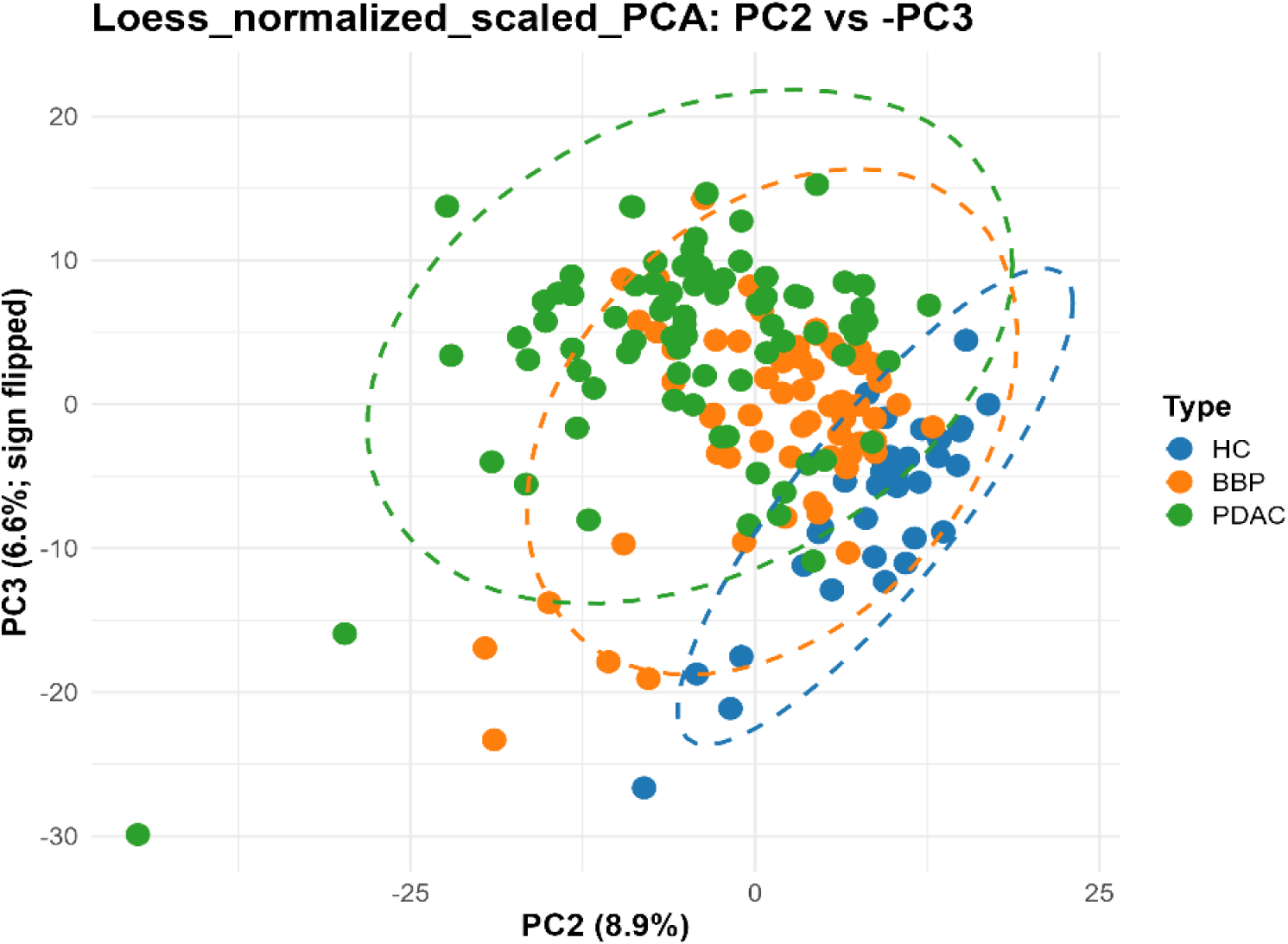
PCA of HC, BBP and PDAC protein profiles. The plot reveals distinct clustering patterns in PC2 and PC3. The BBP samples occupy an intermediate position, overlapping with both PDAC and HC samples.

### 3.3 Dysregulated Proteins in Pancreatic Ductal Adenocarcinoma and Benign Biliary Pathology Samples

Dysregulated proteins were identified by comparing protein abundance levels in PDAC and BBP samples relative to HC. All differential expression analyses were adjusted for age and gender to account for potential demographic confounding effects within the data set. Supporting Information: Differential protein abundance analysis comparing PDAC and BBP samples relative to healthy controls (HC), including complete lists of dysregulated proteins, age- and gender-adjusted differential expression results, and summaries of retained, gained, and lost significance following demographic adjustment (Tables S1–S3, S5–S8, and Figure S8).

The volcano plots in Figure 3 illustrate the distribution of differentially abundant proteins across the three comparisons, highlighting distinct alterations in proteome abundance associated with PDAC, BBP, and HC, using a significance threshold of ≥2-fold. The comparison of BBP vs HC reveals fewer significantly dysregulated proteins, with 12 upregulated and 12 downregulated. In the PDAC vs BBP comparison, substantial molecular differences are observed, with 49 proteins downregulated and 26 upregulated, indicating broader changes between malignant PDAC and benign biliary pathology. The PDAC vs HC comparison shows the most pronounced proteomic dysregulation, with 71 proteins downregulated and 72 upregulated.

**Figure 3:**
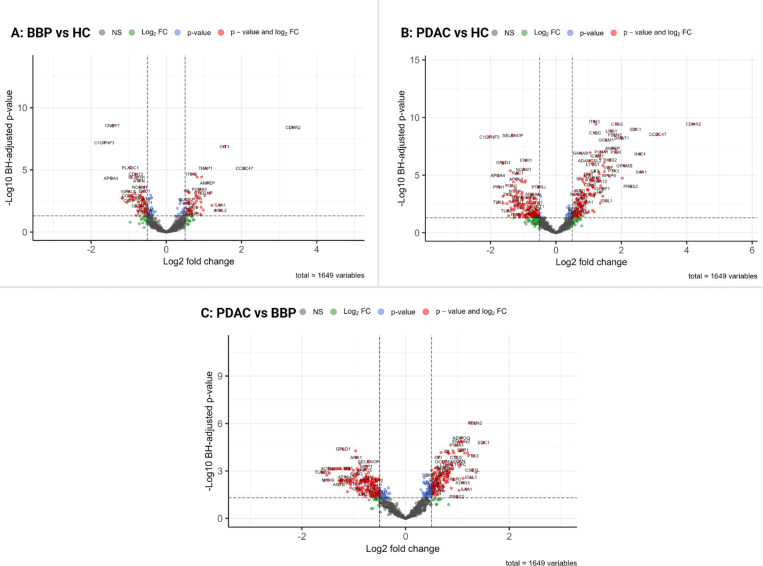
Volcano plots of differentially expressed proteins following age and gender adjustment.

Volcano plots showing differentially expressed proteins in the (A) BBP vs HC, (B) PDAC vs HC and (C) PDAC vs BBP comparisons following sensitivity analysis using a limma model adjusted for age and gender. Proteins are plotted according to their log2 fold change and statistical significance (−log10 adjusted p-value). Proteins with an adjusted p-value < 0.05 were considered significantly differentially expressed.

The protein identifiers were converted into their corresponding gene names and further analyzed using Enrichr,^20–22^ focusing on the MSigDB Hallmark gene sets, to identify enriched cancer-related pathways and determine whether our identified proteins were part of these hallmark gene signatures (Table 2). Using the filtered list of dysregulated proteins comparing PDAC to HC, overrepresentation in several key biological processes was identified. These included Epithelial-Mesenchymal Transition (EMT), with proteins such as PM4, ITGB3, THBS2, FBLN2, VCAN, ANPEP, SPP1, SDC1, PTX3, TGFBI, PRSS2, and MXRA5, supporting their well-established role in promoting tumor invasion and metastasis in PDAC. Additional significantly enriched pathways included apical junction, coagulation, complement, glycolysis, hypoxia, and UV response downregulated signatures, indicating dysregulation of cell adhesion, immune and inflammatory responses, metabolic adaptation, and tumor-associated stress response pathways. As expected, no hallmark pathways were significantly enriched in the comparison between BBP and HC (Table 2).

**Table 2:** Pathway-Level Hallmark Enrichment across PDAC, HC, and BBP Comparisons^a^

Term	p-Value	Adjusted p-Value	Genes
PDAC vs HC			
Epithelial-Mesenchymal Transition	4.95 × 10^–15^	1.98 × 10^–13^	TPM4; ITGB3; THBS2; FBLN2; FBLN5; DCN; CDH6; EFEMP2; VCAN; ANPEP; SPP1; SDC1; FLNA; PTX3; TGFBI; PRSS2; MYL9; MXRA5
Apical Junction	1.10 × 10^–12^	2.20 × 10^–11^	CAP1; VWF; ACTN2; ACTN1; ACTG1; ICAM1; CDH6; VCAN; ZYX; MYH9; MADCAM1; RSU1; TGFBI; PFN1; MYL9; VCL
Coagulation	6.81 × 10^–06^	9.08 × 10^–05^	C8G; WDR1; VWF; ITGB3; CFI; PLEK; CD9; RAC1
Complement	1.40 × 10^–05^	1.40 × 10^–04^	ACTN2; PLEK; S100A12; APOA4; PFN1; S100A9; CTSS; PSMB9; C1QC
Glycolysis	5.98 × 10^–04^	4.79 × 10^–03^	VCAN; PKM; P4HA1; MDH2; SDC1; TGFBI; DCN
Hypoxia	3.19 × 10^–03^	2.13 × 10^–02^	IGFBP1; SRPX; P4HA1; MYH9; TGFBI; DCN
Ultraviolet Response Downregulated	3.79 × 10^–03^	2.16 × 10^–02^	ITGB3; TFPI; LDLR; FBLN5; MGLL
PDAC vs BBP			
Epithelial-Mesenchymal Transition	4.97 × 10^–13^	1.64 × 10^–11^	TPM4; ITGB3; TPM2; THBS2; FBLN2; VCAN; SPP1; FLNA; SDC1; PTX3; PRSS2; MYL9; MXRA5
Apical Junction	1.08 × 10^–11^	1.78 × 10^–10^	CAP1; VCAN; VWF; ACTN2; ACTN1; ZYX; MYH9; RSU1; PFN1; MYL9; ACTB; ACTG1
Myogenesis	9.31 × 10^–04^	1.02 × 10^–02^	ACTN2; TPM3; TPM2; MYH9; MYH11
E2F Targets	6.79 × 10^–03^	4.43 × 10^–02^	TFRC; CSE1L; TUBB; NAP1L1
Heme Metabolism	6.79 × 10^–03^	4.43 × 10^–02^	TFRC; SLC4A1; SPTB; ANK1
Angiogenesis	8.05 × 10^–03^	4.43 × 10^–02^	VCAN; SPP1

a Abbreviations: BBP, Benign biliary pathologies; PDAC, Pancreatic ductal adenocarcinoma; HC, Healthy controls.

To further evaluate molecular pathways associated with PDAC and BBP, within Enrichr.^20–22,30^ The KEGG pathway enrichment analysis was performed using the same list of dysregulated proteins identified from the pairwise comparisons: PDAC vs HC; PDAC vs BBP, and BBP vs HC (Table 3). In the PDAC vs HC comparison, the most significantly enriched pathways were associated with cell adhesion, cytoskeletal organization, extracellular matrix interactions, and platelet-related signaling. Integrin signaling emerged as the most significantly enriched pathway, involving proteins such as VWF, ACTN1, ITGB3, ITGA2B, ILK, THBS2, FLNA, TLN1, and FERMT3. It was followed by the cytoskeleton in muscle cells and focal adhesion pathways, highlighting proteins associated with cytoskeletal remodeling and migratory processes, including TPM4, ACTN2, MYH9, MYL9, VCL, and ZYX. Additional enrichment of platelet activation and ECM-receptor interaction pathways further supported the involvement of platelet-associated signaling, extracellular matrix remodeling, and tumor progression mechanisms in PDAC.

**Table 3:** Enriched KEGG Pathways across PDAC, HC, and BBP Comparisons^a^

Term	p-Value	Adjusted p-Value	Genes
**PDAC vs HC**			
Integrin Signaling	2.96 × 10^–13^	5.15 × 10^–11^	VWF; ACTN1; ITGB3; ITGA2B; ILK; PARVB; THBS2; ACTG1; ICAM1; FLNA; MADCAM1; TLN1; VCL; FERMT3; LIMS1
Cytoskeleton in Muscle Cells	9.31 × 10^–12^	8.10 × 10^–10^	TPM4; ACTN2; ITGB3; ITGA2B; THBS2; FBLN2; DCN; ACTG1; PDLIM1; VCAN; ZYX; SDC1; MYH9; TLN1; MYL9; VCL
Focal Adhesion	1.95 × 10^–09^	1.13 × 10^–07^	VWF; ACTN1; ITGB3; ITGA2B; ILK; PARVB; THBS2; ACTG1; ZYX; FLNA; TLN1; MYL9; VCL
Platelet Activation	2.24 × 10^–08^	9.74 × 10^–07^	STIM1; VWF; GP1BB; GNAQ; ITGB3; ITGA2B; TLN1; GP5; ACTG1; FERMT3
Ecm-Receptor Interaction	3.23 × 10^–06^	1.12 × 10^–04^	VWF; GP1BB; ITGB3; ITGA2B; SDC1; THBS2; GP5
**PDAC vs BBP**			
Cytoskeleton in Muscle Cells	3.38 × 10^–19^	3.59 × 10^–17^	TPM4; ACTN2; TPM3; ITGB3; TPM2; ITGA2B; THBS2; HSPG2; FBLN2; ACTG1; PDLIM1; VCAN; ZYX; MYH9; SDC1; MYH11; TLN1; MYL9
Integrin Signaling	4.48 × 10^–13^	2.37 × 10^–11^	VWF; ITGB3; ACTN1; ITGA2B; ILK; FLNA; PARVB; TLN1; THBS2; ACTG1; FERMT3; LIMS1
Motor Proteins	5.90 × 10^–12^	2.09 × 10^–10^	MYL6; TPM4; TPM3; TUBB; TPM2; MYH9; MYH11; TUBB4B; MYL9; TUBA4A; ACTG1; TUBA8
Focal Adhesion	1.02 × 10^–11^	2.70 × 10^–10^	VWF; ITGB3; ACTN1; ITGA2B; ZYX; ILK; FLNA; PARVB; TLN1; THBS2; MYL9; ACTG1
Igsf Cam Signaling	9.31 × 10^–10^	1.70 × 10^–08^	MYL6; ITGB3; ACTN1; TUBB; MYH9; MYH11; F11R; TUBB4B; MYL9; TUBA4A; ACTG1; TUBA8
**BBP vs HC**			
Cholesterol Metabolism	3.84 × 10^–07^	1.04 × 10^–05^	CETP; APOA2; APOA4; LPA
Fat Digestion and Absorption	0.001209	1.63 × 10^–02^	MTTP; APOA4

a Abbreviations: BBP, Benign biliary pathologies; PDAC, Pancreatic ductal adenocarcinoma; HC, Healthy controls.

The PDAC vs BBP comparison demonstrated a similar but more pronounced enrichment profile, with the cytoskeleton in muscle cells identified as the most significantly enriched pathway. This pathway included multiple structural and contractile proteins, including TPM4, TPM3, TPM2, ACTN2, MYH9, MYH11, MYL9, and TLN1, suggesting substantial cytoskeletal reorganization associated with malignant transformation. Integrin signaling and focal adhesion pathways were again significantly enriched, involving proteins related to adhesion and extracellular matrix regulation, including ITGB3, ACTN1, ILK, FLNA, PARVB, THBS2, and FERMT3. Additional enrichment of motor proteins and IGSF CAM signaling pathways reflected alterations in intracellular transport, cellular motility, and adhesion-associated communication processes.

The comparison between BBP and HC primarily highlighted metabolic processes. Cholesterol metabolism was again the most significantly enriched, involving CETP, APOA2, APOA4, and LPA, while fat digestion and absorption pathways were also enriched through proteins such as MTTP and APOA4.

An UpSet plot was generated to illustrate the set sizes and highlight both the unique and overlapping dysregulated proteins across the three comparative groups: PDAC vs HC, PDAC vs BBP, and BBP vs HC (Figure 4). The PDAC vs HC comparison showed the largest number of proteins, with 69 proteins (42.3%) exclusive to this group. Seventeen proteins (10.4%) were uniquely identified in the PDAC, while three proteins (1.8%) were unique to the BBP vs HC comparison. Additionally, bar plots were included in Figure 4 to visualize the directionality (upregulation or downregulation) of the overlapping protein sets, allowing assessment of their potential utility in monitoring progressive disease-associated proteomic changes. A total of 53 proteins (32.5%) were found to overlap between the PDAC vs HC and PDAC vs BBP comparisons. This finding supports enrichment results related to integrin signaling, focal adhesion, cytoskeletal remodeling, platelet activation, extracellular matrix organization, and EMT, as identified in the pathway analyses. Additionally, 16 overlapping proteins (9.8%) were identified between the BBP vs HC and PDAC vs HC comparisons, which were also included in the enriched pathway. These proteins were primarily associated with cholesterol metabolism, fat digestion and absorption, complement-related processes, inflammatory regulation, and EMT pathways. Five proteins (3.1%) were common across all three comparisons. These included PRSS2, which is directly associated with the enriched EMT pathway; GPNMB, implicated in tumor progression; and SAA1, a well-established acute-phase inflammatory protein. To delineate the protein signatures of PDAC aggressiveness, Spearman’s test was performed to link protein concentration values to the different groups. LRG1 showed the strongest association with disease severity (Figure 5). ROC analysis yielded an AUC of 0.94 (95% CI: 0.88–0.99) for discriminating HC from PDAC, and 0.85 (95% CI: 0.78–0.91) for discriminating BBP from PDAC. To explore coordinated patterns in protein concentrations across the 178 patient samples, KODAMA was applied to the correlation distance matrix of quantified proteins. This approach allows the identification of groups of proteins that share similar correlation profiles, suggesting potential coregulation or corelease mechanisms. By projecting the correlation structure in a low-dimensional manifold and performing graph-based clustering, different protein communities, characterized by high intracluster correlation, were identified. These clusters likely represent functional modules of proteins that are simultaneously released into the bloodstream, potentially reflecting the activity of specific cell types or biological processes within the tissue origin. The correlation between the proteins is illustrated in Figure 6. Notably, LRG1 clusters with several proteins that are positively associated with PDAC, including C1QC, CCDC47, CDHR2, ORM1, PRSS2, and SAA1.

**Figure 4:**
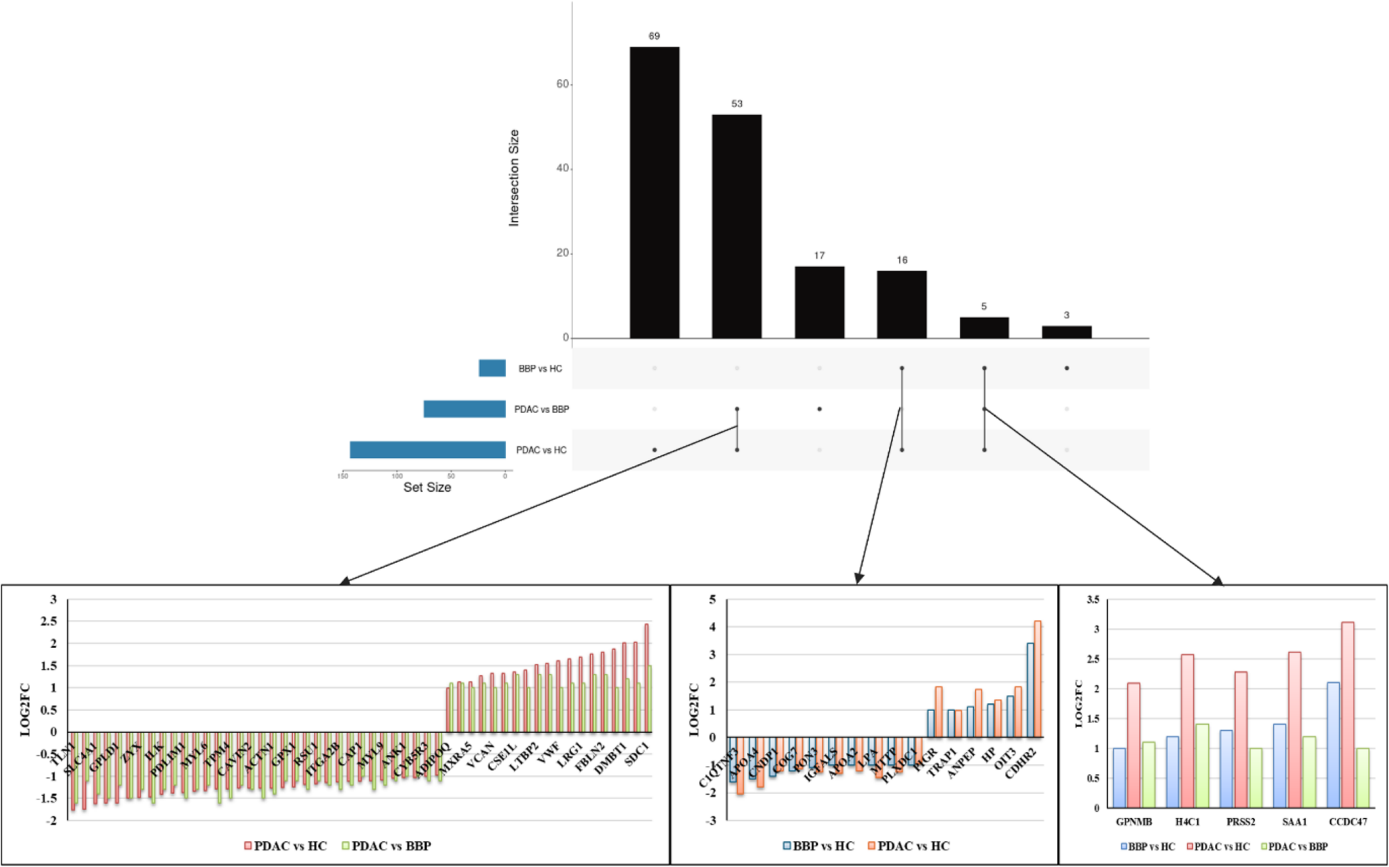
UpSet plot illustrating the unique and overlapping dysregulated proteins across the three comparative groups, together with bar plots showing the log_2_fold-change directionality of overlapping proteins. UpSet plot showing the number of unique and shared dysregulated proteins (black bars) and the total set size (blue bars) for each comparison group (PDAC vs BBP, PDAC vs HC, and BBP vs HC). Bar plots depicting the log_2_ fold-change values of proteins shared between the comparison groups. Negative values indicate downregulated proteins, while positive values indicate upregulated proteins.

**Figure 5:**
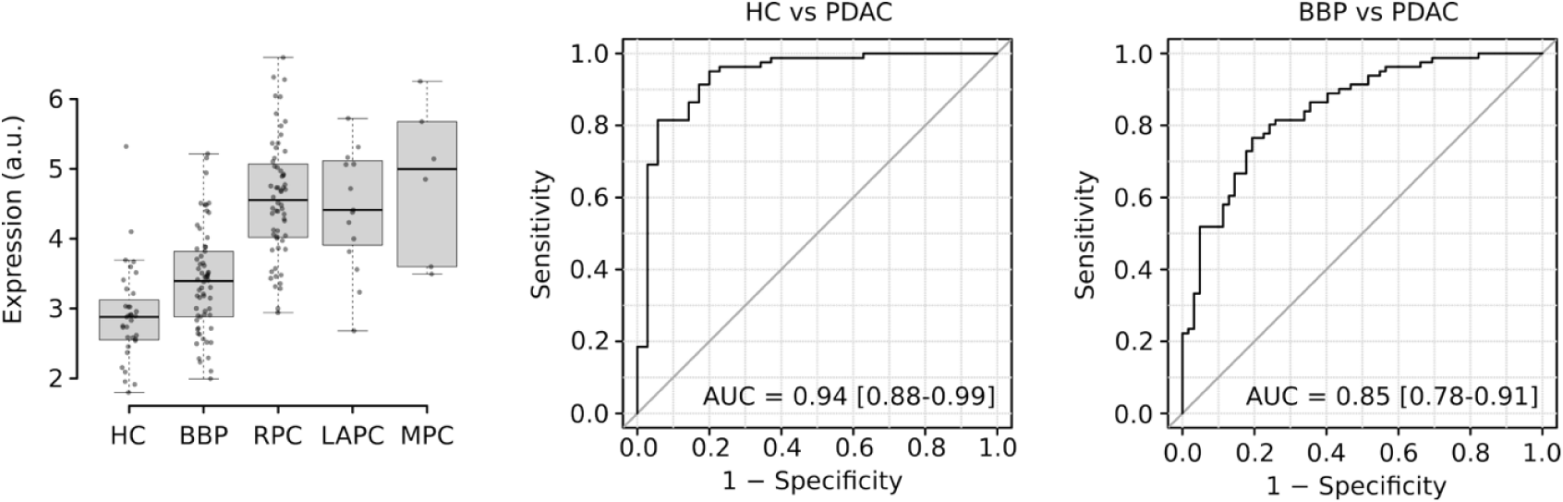
Association of LRG1 protein levels with disease severity. The boxplot shows LRG1 protein levels across all groups (HC, BBP, RPC, LAPC, and MPC). The ROC curves illustrate the ability of LRG1 to discriminate HC vs PDAC and BBP vs PDAC. AUC values and relative 95% CI are reported.

**Figure 6:**
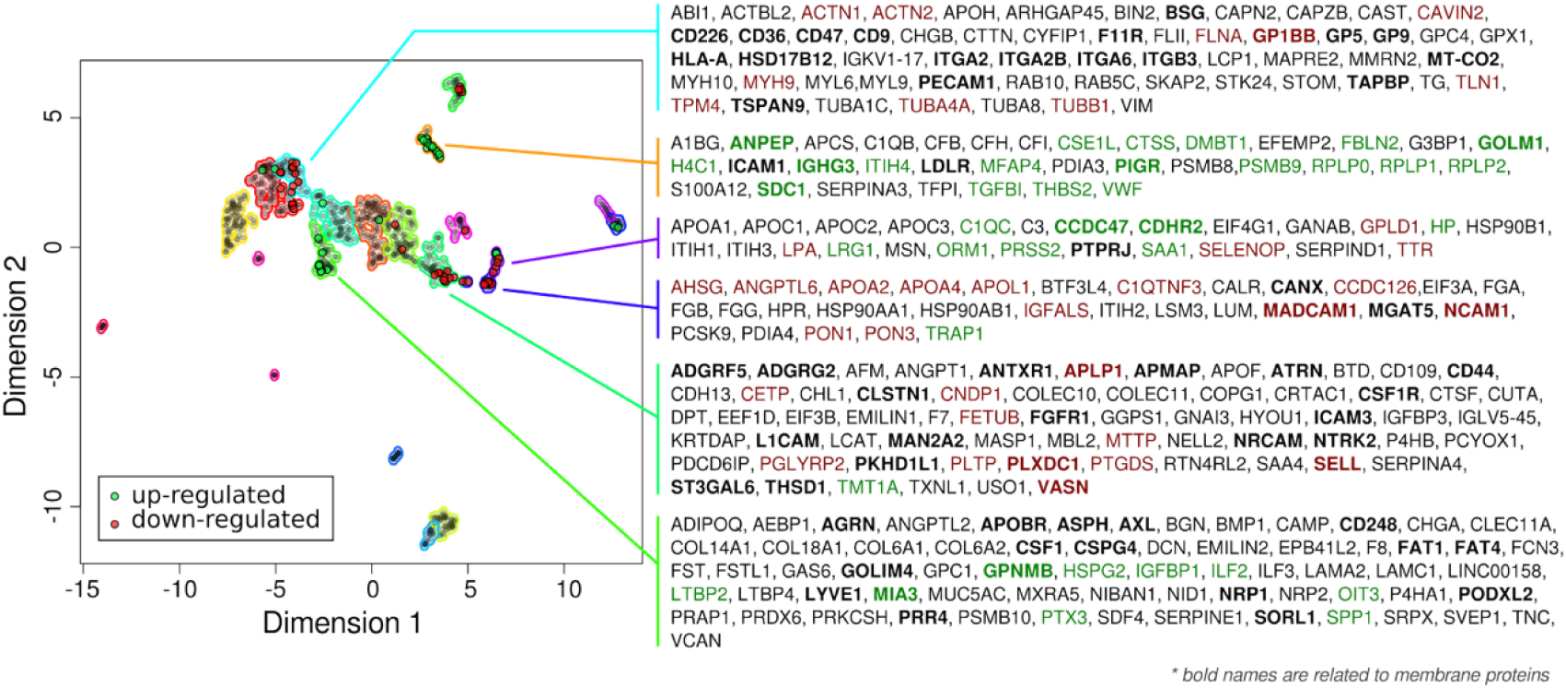
Protein correlation map. KODAMA analysis was used to highlight clusters of proteins exhibiting strong correlations. The different colors indicate distinct protein clusters with the membrane proteins in bold.

## 4 Discussion

This study compared the plasma proteome of patients with PDAC with that of noncancer controls (HC and BBP). The BBP subgroup was included in the study because it has been reported to mimic many features of PDAC. Both conditions can lead to cholestasis and systemic inflammation, and they exhibit similar clinical presentations, such as jaundice, abdominal pain, weight loss, and abnormal liver function tests. This overlap was further supported by observations of progressive increases in CRP, ALP, and GGT with increasing disease severity. These shared biochemical abnormalities underscore the diagnostic challenge of distinguishing malignant from benign biliary obstruction, which often leads to misdiagnosis or delayed diagnosis of PDAC.^31–35^

Mass spectrometry-based label-free quantification was used to detect distinct patterns of protein dysregulation that enabled clustering of the proteomic profiles among these three groups. The plasma proteome may provide additional discriminatory and severity-related value in the clinical assessment of PDAC. The enrichment analysis data revealed that PDAC is characterized by disruptions in cell adhesion, extracellular matrix remodeling, and cytoskeletal organization. In contrast, BBP exhibited metabolic alterations, particularly in lipid-handling and digestion-related pathways. In contrast, BBP vs HC exhibited a more limited enrichment profile, with metabolic alterations restricted to cholesterol metabolism and fat digestion and absorption pathways. suggesting metabolic reprogramming in benign conditions. No hallmark pathways were significantly enriched in BBP relative to HC, consistent with the expectation that hallmark-level oncogenic processes are predominantly active in malignant rather than benign biliary states.

The comparative analysis of PDAC vs HC, PDAC vs BBP, and BBP vs HC provides insights into proteins that are significantly dysregulated and are nonoverlapping or overlapping across the group comparisons.

In comparison between PDAC and HC, a majority of the downregulated proteins were associated with cytoskeletal organization, platelet activation, lipid metabolism, antioxidant defense, and cellular transport processes. Notable examples of these proteins include SELENOP, AHSG, RAC1, VCL, CFL1, and PRDX6, many of which play crucial roles in lipid handling, redox balance, and the maintenance of cellular structural integrity.^36–40^ The reduced abundance of proteins linked to lipid metabolism and antioxidant pathways has been previously reported in PDAC. This reduction may reflect tumor-driven metabolic reprogramming, as well as systemic inflammatory responses associated with pancreatic tumor progression.^40,41^

The upregulated proteins were mainly linked to inflammatory signaling, extracellular matrix remodeling, complement activation, hypoxia, immune modulation, and tumor progression. Proteins such as TGFBI, ICAM1, S100A9, S100A12, EFEMP2, IGFBP1, GOLM1, CAMP, FCGBP, and LYVE1 have been previously associated with tumor invasion, stromal activation, angiogenesis, and inflammatory responses in PDAC. Many of these proteins, including GOLM1, TGFBI, S100A9, and ICAM1, have also been connected to aggressive PDAC progression and poor prognosis in international studies, highlighting the broader biological significance of these findings.^42–46^ Similarly, increased abundance of complement-associated proteins such as C1QB, C1QC, CFI, and C8G supports activation of innate immune and inflammatory signaling within the PDAC tumor microenvironment, while extracellular matrix-associated proteins, including TGFBI, EFEMP2, DCN, and MFAP4, are consistent with the highly desmoplastic nature of pancreatic tumors.^47–50^

The proteins dysregulated in the PDAC vs BBP comparison were predominantly downregulated and included those linked to cytoskeletal organization, intracellular trafficking, structural integrity, and adhesion. Key proteins included ACTB, MYH11, TPM2, and others involved in actin filament dynamics and microtubule organization. This dysregulation may contribute to increased tumor cell plasticity and invasiveness.^51,52^ Additional downregulated proteins, such as F11R and RAB27B, indicate disrupted epithelial integrity and adhesion signaling. Conversely, only HSPG2 and TFRC were upregulated in PDAC; HSPG2 is linked to tumor-stromal interactions, and TFRC is associated with increased metabolic demand in tumor cells.^53,54^

The overlapping protein profiles suggest that PDAC and BBP share some inflammatory and metabolic changes; however, PDAC exhibits a stronger and broader tumor-associated signature. Five proteins that were consistently upregulated across all comparisons- GPNMB, H4C1, PRSS2, SAA1, and CCDC47- showed the highest fold changes when comparing PDAC to healthy controls. This finding is significant because it indicates a progressive increase in protein levels from benign biliary conditions to malignant disease, suggesting these proteins as potential markers of disease progression. Additionally, this pattern aligns with previous studies linking SAA1 to inflammation and a poor prognosis in pancreatic cancer, while GPNMB has been associated with tumor progression and immune modulation.^55,56^

The proteins overlapping with PDAC vs HC and PDAC vs BBP showed a clearer PDAC-specific pattern. The first group showed downregulation in lipid metabolism and transport, including apolipoproteins and lipid transport mediators (e.g., APOA2, APOA4, MTTP, PON3, LPA). These observations support the view that lipid homeostasis is disrupted in both disease states but is amplified in malignancy.^57–60^ This aligns with earlier studies on pancreatic cancer’s metabolomics and lipoproteomics, indicating disrupted lipid metabolism and systemic metabolic disturbances in PDAC.^61^ The proteins overlapping with PDAC vs HC and PDAC vs BBP analysis showed a clearer PDAC-specific pattern. For instance, downregulated proteins such as TLN1, PFN1, ACTN2, ZYX, ILK, FERMT3, FLNA, TPM4, MYH9, ITGB3, ITGA2B, GP1BB, and MYL9 were largely associated with cytoskeletal organization, integrin signaling, focal adhesion, and platelet-related processes. In contrast, upregulated proteins such as SPP1, VCAN, THBS2, PTX3, FBLN2, LRG1, CTSS, DMBT1, and SDC1 were associated with extracellular matrix remodeling, stromal activation, inflammation, and epithelial-mesenchymal transition.^62–66^ What stands out most is the consistent directionality of the overlapping proteins and the stronger magnitude of change observed in the PDAC vs HC comparison, particularly for CCDC47, SAA1, H4C1, PRSS2, GPNMB, CDHR2, SDC1, DMBT1, CTSS, FBLN2, PTX3, LRG1, and THBS2. Notably, LRG1 showed the strongest association with disease severity, supporting previous reports of its pro-tumorigenic role in PDAC, including promotion of tumor progression, angiogenesis, and modulation of the tumor microenvironment.^67^ It also clustered with several proteins positively associated with PDAC, including C1QC, CCDC47, CDHR2, ORM1, PRSS2, and SAA1, supporting a coordinated inflammation-stromal remodeling program characteristic of aggressive disease.^68–70^ LRG1 has also Notably, this cluster also includes the membrane protein PTPRJ, a receptor-type tyrosine phosphatase commonly expressed by innate immune cells, particularly myeloid lineages.^71^ Together, these associations support a shared biological origin driven by innate immune activation, potentially mediated, at least in part, by immune cell-derived extracellular vesicles that contribute to the circulating protein signature.

## 5 Conclusion

Overall, the findings show that while PDAC shares significant clinical and biochemical characteristics with BBP, plasma proteomic profiling can detect distinct molecular signatures that distinguish malignant disease from nonmalignant conditions. The observed proteomic patterns further suggest that PDAC within this cohort is distinguished by amplified stromal activation, extracellular matrix remodeling, inflammatory signaling, and cytoskeletal dysregulation, whereas BBP demonstrates comparatively modest metabolic and inflammatory alterations. Although these findings are broadly consistent with international PDAC proteomic studies, the strong inflammatory, stromal, and metabolic signatures observed in this South African cohort highlight the importance of evaluating PDAC biology in underrepresented African populations, where environmental, genetic, and clinical heterogeneity may influence disease presentation and biomarker profiles.

Collectively, these results indicate that plasma proteomics provides discriminatory and disease severity-related insights beyond conventional clinical biomarkers. While several candidate proteins show promise for incorporation into multianalyte biomarker panels, further validation in larger, independent, and statistically powered African cohorts will be essential to establish their clinical utility for early detection, risk stratification, and improved differential diagnosis of PDAC.

## Supplementary Material



## Data Availability

The mass spectrometry proteomics (raw) data, along with the output file from Spectronaut 19, have been deposited to the ProteomeXchange Consortium via the PRIDE^72^ partner repository with the data set identifier: PXD074950.

## Notes

Table S1: list of dysregulated proteins in PDAC compared with HC; Table S2: list of dysregulated genes in PDAC compared with BBP; Table S3: list of dysregulated genes in BBP compared with HC; Figure S1: bar graphs indicating the number of protein groups and modified peptides identified in each HeLa_QC replicate; Figure S2: bar graphs indicating the number of protein groups and modified peptides identified in each Pool_QC replicate; Figure S3: multiscatter plots showing Pearson correlation of identified protein groups between the HeLa_QC replicates; Figure S4: multiscatter plots showing Pearson correlation of identified peptides between the HeLa_QC replicates; Figure S5: multiscatter plots showing Pearson correlation of identified protein groups between the Pool_QC replicates; Figure S6: multiscatter plots showing Pearson correlation of identified peptides between the Pool_QC replicates; Figure S7: variability between QC replicates; Table S4: table of LC-derived peak parameters; Figure S8: bar and box plots indicating gender and age distribution for the PDAC, BBP, and HC patient cohort; Table S5: summary of differential protein significance changes following age and gender adjustment across the PDAC vs HC, PDAC vs BBP, and BBP vs HC comparisons; Table S6: list of dysregulated proteins in BBP compared with HC post gender and age adjustment; Table S7: list of dysregulated proteins in PDAC compared with HC post gender and age adjustment; Table S8: list of dysregulated proteins in PDAC compared with BBP post gender and age adjustment; Figure S9: PCA of HC, BBP, and PDAC protein profiles (PDF)
