# Supplementary material for: Proteomic Profiling of Extracellular Vesicle-Enriched Plasma Using Mag-Net for Biomarker Discovery in Pancreatic Ductal Adenocarcinoma
Source: J Proteome Res. 2026 Jun 30;25(8):4120–31. doi: 10.1021/acs.jproteome.6c00172 (PMC13459540; doi:10.1021/acs.jproteome.6c00172)
Supplement: Supplementary file 1 [file pr6c00172_si_001.pdf]

Proteomic profiling of extracellular vesicle-enriched plasma using Mag-Net for biomarker discovery in pancreatic ductal adenocarcinoma

Sindisiwe Buthelezi[1], Nnenna Elebo[2], Previn Naicker[3], Rethabile Mokoena[1], Sinegugu Dubazana[2], Ireshyn Govender[1], Sipho Mamputha[1], Andrea Ellero[4], Stoyan Stoychev[5], Jeanet Mazibuko[2], Dupe Ojo[3], Geoffrey Candy[2], John Devar[2,6], Stefano Cacciatore[7,8,] Jones Omoshoro-Jones[2,6] and Ekene Emmanuel Nweke[2,9]*

[1] Future Production: Chemicals Cluster, Council for Scientific and Industrial Research, Pretoria, 0001, South Africa

[2] Department of Surgery, Faculty of Health Sciences, University of Witwatersrand, Johannesburg 2193, South Africa.

[3] ReSyn Biosciences, Gauteng, South Africa

[4] Department of Pharmacology, Faculty of Health Sciences, University of Pretoria, Pretoria 0007, South Africa

[5] Evosep Biosystems, Billedskærervej 15, 5230 Odense, Denmark.

[6] Hepatopancreatobiliary Unit, Department of Surgery, Chris Hani-Baragwanath Academic Hospital, Soweto, Johannesburg, South Africa.

[7] Department of Integrative Biomedical Sciences, Faculty of Health Sciences, University of Cape Town, South Africa.

[8] Bioinformatics Unit, International Centre for Genetic Engineering and Biotechnology, Observatory 7925, Cape Town, South Africa.

[9] Department of Life and Consumer Sciences, College of Agriculture and Environmental Sciences, University of South Africa, Florida, Roodepoort, South Africa.

*Corresponding author: Ekene Nweke (ekene.nweke@wits.ac.za)

Contents

1. List of dysregulated proteins 3

Table S1. List of dysregulated proteins in PDAC compared with HC. 3

Table S2. List of dysregulated genes in PDAC compared with BBP. 5

Table S3. List of dysregulated genes in BBP compared with HC 6

2. Quality control (QC) for PDAC study 6

Figure S1: Bar graphs indicating the number of protein groups and modified peptides identified in each Hela_QC replicate. 8

Figure S2: Bar graphs indicating the number of protein groups and modified peptides identified in each Pool_QC replicate. 9

Figure S3: Multi-scatter plots showing Pearson correlation of identified protein groups between the Hela_QC replicates. 10

Figure S4: Multi-scatter plots showing Pearson correlation of identified peptides between the Hela_QC replicates 11

Figure S5: Multi-scatter plots showing Pearson correlation of identified protein groups between the Pool_QC replicates 12

Figure S6: Multi-scatter plots showing Pearson correlation of identified peptides between the Pool_QC replicates 13

Figure S7: Variability between QC replicates 14

Table S4: Table of LC derived peak parameters 16

3. List of dysregulated proteins post gender and age adjustment 17

Figure S8: Bar and box blots indicating gender and age distribution for the PDAC, BBP and HC patient cohort. 17

Table S5: Summary of differential protein significance changes following age and gender adjustment across the PDAC vs HC, PDAC vs BBP, and BBP vs HC comparisons. 18

Table S6: List of dysregulated proteins in BBP compared with HC post gender and age adjustment. 18

Table S7: List of dysregulated proteins in PDAC compared with HC post gender and age adjustment. 20

Table S8: List of dysregulated proteins in PDAC compared with BBP post gender and age adjustment. 27

4. PCA projection 31

Figure S9: PCA of HC, BBP and PDAC protein profiles. 31

1. List of dysregulated proteins

Tables S1-3 list dysregulated proteins identified in the comparison among patients with pancreatic ductal adenocarcinoma (PDAC), those with benign biliary pathologies (BBP), and healthy controls (HC). These tables include the corresponding log-fold change values and statistical significance measures. Positive log-fold-change values indicate proteins that are upregulated, while negative values indicate proteins that are downregulated.

Table S1. List of dysregulated proteins in PDAC compared with HC.

Gene name	log2FC	AveExpr	t	*p*-value	Adjusted *p*-value	B
APOA4	-1.93	9.613011944	-7.99144	1.49E-13	8.7874E-12	20.28877
LPA	-1.87	8.668019881	-4.64015	6.64E-06	4.47645E-05	3.203845
C1QTNF3	-1.87	4.847446761	-8.89814	5.66E-16	8.12073E-14	25.74313
IGFALS	-1.84	7.357513117	-9.2453	6.32E-17	1.26809E-14	27.89302
MADCAM1	-1.81	8.073070736	-8.98593	3.26E-16	5.45618E-14	26.2839
GPLD1	-1.71	8.560298556	-8.69054	2.07E-15	2.31024E-13	24.47261
PON1	-1.62	7.386350512	-7.64283	1.18E-12	4.94033E-11	18.26399
PON3	-1.51	5.158121732	-6.2044	3.61E-09	6.04458E-08	10.44703
AHSG	-1.50	7.995521437	-8.14505	5.89E-14	3.94483E-12	21.1946
MTTP	-1.42	3.795928146	-6.80053	1.45E-10	3.38806E-09	13.57147
APOA2	-1.39	5.588503234	-6.71086	2.38E-10	5.08172E-09	13.09032
PFN1	-1.36	8.209795822	-4.83542	2.82E-06	2.11237E-05	4.023014
CNDP1	-1.36	5.136231771	-8.39069	1.32E-14	1.10385E-12	22.65935
SELENOP	-1.36	8.869339388	-9.99629	5.01E-19	1.67682E-16	32.63715
CETP	-1.35	5.209067574	-5.99084	1.09E-08	1.66484E-07	9.37245
COG7	-1.32	9.27058184	-5.39089	2.16E-07	2.22849E-06	6.491947
PTGDS	-1.29	6.614489987	-6.59571	4.46E-10	9.14566E-09	12.47814
APOL1	-1.24	9.61996876	-6.99579	4.88E-11	1.29043E-09	14.63205
ANGPTL6	-1.18	8.594162058	-7.44769	3.69E-12	1.27858E-10	17.15056
VASN	-1.14	5.397799713	-7.50834	2.6E-12	9.30534E-11	17.49505
ENO3	-1.13	6.304505656	-4.46084	1.43E-05	8.84586E-05	2.475112
TMSB4X	-1.12	6.158851526	-4.23579	3.61E-05	0.000207314	1.593188
PLXDC1	-1.11	4.089783209	-7.95763	1.82E-13	1.01649E-11	20.09048
TAGLN2	-1.09	5.850066855	-4.56988	8.99E-06	5.89819E-05	2.915522
PGLYRP2	-1.08	5.922520996	-5.59366	8.06E-08	9.86431E-07	7.441788
NCAM1	-1.06	9.286288585	-8.31614	2.08E-14	1.6081E-12	22.21273
FETUB	-1.05	5.906590102	-6.1997	3.7E-09	6.09356E-08	10.42317
APLP1	-1.04	7.479457829	-6.32032	1.96E-09	3.64367E-08	11.04054
LDHB	-1.02	5.59441169	-4.77534	3.68E-06	2.639E-05	3.768164
TTR	-0.99	10.35505216	-5.38912	2.18E-07	2.22849E-06	6.483793

CCDC126	-0.99	3.410356504	-5.41233	1.95E-07	2.05639E-06	6.591203
PLTP	-0.98	5.804290985	-5.17912	5.88E-07	5.46357E-06	5.52717
SELL	-0.98	7.046098729	-6.22047	3.32E-09	5.74696E-08	10.52891
TMT1A	0.97	4.431111551	5.47077	1.47E-07	1.63711E-06	6.863149
ITIH4	0.99	7.960800838	6.353437	1.64E-09	3.11343E-08	11.21139
PSMB9	0.99	5.612752128	6.19518	3.79E-09	6.13919E-08	10.40015
TGFBI	1.00	8.547246573	5.601683	7.75E-08	9.72004E-07	7.479854
C1QC	1.01	6.396165291	8.611948	3.37E-15	3.38666E-13	23.99478
ILF2	1.06	3.699225886	5.681634	5.22E-08	6.98369E-07	7.861526
TRAP1	1.09	8.615108455	6.988681	5.08E-11	1.30861E-09	14.5931
SPP1	1.09	6.865206701	5.837583	2.39E-08	3.47599E-07	8.616743
MIA3	1.10	5.842013819	5.964803	1.25E-08	1.87427E-07	9.24312
ORM1	1.11	8.613162576	6.387409	1.37E-09	2.64778E-08	11.38723
IGLV1-47	1.11	8.575870165	5.161219	6.39E-07	5.83131E-06	5.44692
OIT3	1.14	6.322135753	5.434416	1.75E-07	1.88863E-06	6.693735
THBS2	1.18	7.348086107	5.806411	2.8E-08	4.00961E-07	8.464667
HP	1.20	7.108342444	5.403097	2.03E-07	2.12732E-06	6.548432
RPLP1	1.21	6.695738429	5.673262	5.44E-08	7.18416E-07	7.821384
IGFBP1	1.25	6.463044103	5.614747	7.26E-08	9.22989E-07	7.541963
GOLM1	1.28	8.924868604	7.884545	2.82E-13	1.41622E-11	19.66321
IGLL1	1.29	6.097949183	4.442474	1.54E-05	9.49651E-05	2.401742
ANPEP	1.31	5.446458994	6.815929	1.33E-10	3.18528E-09	13.65446
FBLN2	1.32	5.760253766	7.06059	3.39E-11	9.74764E-10	14.98779
LTBP2	1.34	5.217972033	7.760013	5.91E-13	2.6992E-11	18.93963
MFAP4	1.35	4.727468414	5.314615	3.1E-07	3.08592E-06	6.141216
RPLP2	1.36	7.272321407	6.875645	9.56E-11	2.34201E-09	13.97738
VWF	1.38	9.004028282	6.77927	1.63E-10	3.64128E-09	13.45704
CTSS	1.47	5.178136459	8.295994	2.35E-14	1.68863E-12	22.09237
IGHG3	1.50	5.393029738	5.153393	6.63E-07	5.89973E-06	5.411902
GPNMB	1.51	6.466503406	5.682265	5.2E-08	6.98369E-07	7.864554
PTX3	1.52	7.535667928	6.60397	4.27E-10	8.92613E-09	12.52182
DMBT1	1.56	5.443637349	7.358591	6.19E-12	2.00315E-10	16.64723
RPLP0	1.58	4.549684239	7.009882	4.51E-11	1.22438E-09	14.70923
PIGR	1.58	4.651904253	7.717554	7.6E-13	3.3189E-11	18.69423
LRG1	1.73	3.835371518	10.4468	2.62E-20	2.62926E-17	35.53371
SDC1	1.99	8.956659934	8.39884	1.26E-14	1.10385E-12	22.70826
H4C1	2.24	8.365452184	7.597392	1.54E-12	6.0622E-11	18.00341
PRSS2	2.30	5.554212278	6.564374	5.29E-10	1.06258E-08	12.31264
SAA1	2.78	6.504516159	8.11343	7.14E-14	4.47787E-12	21.00747
CCDC47	3.17	6.560945967	10.27698	8E-20	4.01432E-17	34.43803
CDHR2	3.94	7.806342566	9.781026	2.03E-18	5.08922E-16	31.26558

Negative values indicate downregulation in PDAC, while positive values indicate upregulation in PDAC.

Table S2. List of dysregulated genes in PDAC compared with BBP.

Gene name	log2FC	AveExpr	t	*p*-value	Adjusted *p*-value	B
GPLD1	-1.24	8.560299	-7.52419	2.37E-12	7.92E-10	17.6639
TUBB1	-1.20	9.172386	-3.98815	9.64E-05	0.000827	0.898981
PDLIM1	-1.18	7.863113	-4.53635	1.04E-05	0.000134	2.994232
TPM4	-1.17	8.517531	-3.95457	0.00011	0.000911	0.777847
TLN1	-1.17	8.932295	-3.88109	0.000145	0.001114	0.515866
PFN1	-1.15	8.209796	-4.89302	2.18E-06	4.56E-05	4.471871
FLNA	-1.10	10.42126	-3.27236	0.001276	0.005851	-1.49124
ILK	-1.10	7.726218	-4.51849	1.12E-05	0.00014	2.922554
MYH9	-1.09	8.957324	-3.42929	0.000749	0.003797	-1.0023
ACTB	-1.09	10.77708	-3.67056	0.000318	0.002059	-0.21165
ACTN2	-1.07	8.756804	-4.08256	6.67E-05	0.000614	1.244025
ACTN1	-1.06	8.279198	-4.25309	3.37E-05	0.00036	1.884151
PARVB	-1.04	7.075338	-4.59242	8.16E-06	0.000118	3.22074
CAVIN2	-1.01	6.869665	-4.31795	2.58E-05	0.000286	2.133251
TUBA4A	-1.01	9.59055	-3.56099	0.000472	0.002753	-0.57651
TTR	-1.00	10.35505	-6.53003	6.37E-10	7.11E-08	12.26323
PON1	-0.99	7.386351	-5.60692	7.55E-08	3.61E-06	7.678586
FERMT3	-0.97	7.64515	-4.70573	5E-06	8.87E-05	3.685097
GP1BB	-0.96	8.97885	-3.42961	0.000748	0.003797	-1.00128
POSTN	0.97	3.040753	6.25984	2.7E-09	2.08E-07	10.87453
RPLP0	1.00	4.549684	5.291049	3.47E-07	1.03E-05	6.220481
HSPG2	1.00	7.225066	6.8257	1.26E-10	3.17E-08	13.82367
SPP1	1.00	6.865207	6.45746	9.42E-10	8.6E-08	11.88666
FBLN2	1.04	5.760254	6.663638	3.08E-10	4.42E-08	12.96326
CSE1L	1.04	5.286237	4.621512	7.2E-06	0.000111	3.339108
VWF	1.04	9.004028	6.131712	5.28E-09	3.79E-07	10.22904
DMBT1	1.06	5.443637	5.97198	1.21E-08	8.07E-07	9.436561
IGLL1	1.11	6.097949	4.559794	9.38E-06	0.000126	3.088669
CCDC47	1.18	6.560946	4.576871	8.72E-06	0.000122	3.157699
PRSS2	1.18	5.554212	4.035068	8.03E-05	0.00072	1.069606
CDHR2	1.18	7.806343	3.517265	0.000551	0.003071	-0.71941
LTBP2	1.19	5.217972	8.269481	2.77E-14	1.39E-11	21.96835
LRG1	1.21	3.835372	8.771166	1.25E-15	1.26E-12	24.96664
PTX3	1.25	7.535668	6.488364	7.98E-10	8.01E-08	12.04671
H4C1	1.31	8.365452	5.310462	3.17E-07	9.93E-06	6.308339
SDC1	1.33	8.95666	6.721063	2.25E-10	3.76E-08	13.26674
SAA1	1.50	6.504516	5.252933	4.16E-07	1.16E-05	6.048661

Negative values indicate downregulation in PDAC, while positive values indicate upregulation in PDAC.

Table S3. List of dysregulated genes in BBP compared with HC

Gene name	log2FC	AveExpr	t	*p*-value	Adjusted *p*-value	B
C1QTNF3	-1.56707	4.847446761	-7.1367321	2.2E-11	7.37E-09	15.51238
APOA4	-1.54472	9.613011944	-6.1257175	5.45E-09	4.97E-07	10.21433
CNDP1	-1.48146	5.136231771	-8.7375595	1.55E-15	1.55E-12	24.74663
PON3	-1.26103	5.158121732	-4.9616199	1.6E-06	4.22E-05	4.791973
ENO3	-1.18546	6.304505656	-4.496769	1.23E-05	0.000192	2.865391
LPA	-1.18019	8.668019881	-2.7957546	0.005734	0.024709	-2.80992
CETP	-1.15807	5.209067574	-4.9347367	1.81E-06	4.65E-05	4.676592
MTTP	-1.13282	3.795928146	-5.1972379	5.4E-07	2.26E-05	5.823276
IGFALS	-1.06169	7.357513117	-5.1062853	8.24E-07	2.7E-05	5.420951
COG7	-1.05768	9.27058184	-4.1196297	5.76E-05	0.000672	1.413767
PLXDC1	-1.05142	4.089783209	-7.1933511	1.6E-11	7.37E-09	15.82288
PTGDS	-1.04293	6.614489987	-5.1128211	8E-07	2.7E-05	5.449687
PIP	-1.02523	3.65830483	-5.2421284	4.37E-07	2E-05	6.023774
PLTP	-1.02309	5.804290985	-5.1504883	6.72E-07	2.5E-05	5.615825
APOA2	-0.99597	5.588503234	-4.6055113	7.71E-06	0.000136	3.302815
MADCAM1	-0.96	8.073070736	-4.5669143	9.1E-06	0.000152	3.146609
TREML1	0.977169	5.351048036	3.1424207	0.001957	0.010967	-1.84235
HP	0.983665	7.108342444	4.2218333	3.82E-05	0.000474	1.79693
IGLV1-47	0.994162	8.575870165	4.4079358	1.78E-05	0.000255	2.514233
MYH11	1.002965	7.552360682	2.6763391	0.008123	0.032365	-3.1193
FLNA	1.019926	10.42126302	2.4179395	0.016596	0.053372	-3.74585
TRAP1	1.037064	8.615108455	6.3888563	1.36E-09	1.95E-07	11.5456
OIT3	1.113765	6.322135753	5.0956296	8.66E-07	2.72E-05	5.374161
PRSS2	1.1188	5.554212278	3.0599266	0.002549	0.013541	-2.08185
PDIA6	1.135869	7.539584404	3.9858854	9.72E-05	0.001084	0.92411
SAA1	1.279289	6.504516159	3.5699647	0.000457	0.003528	-0.51101
CCDC47	1.991517	6.560945967	6.1795206	4.12E-09	4.13E-07	10.48361
CDHR2	2.755	7.806342566	6.5507182	5.7E-10	1.22E-07	12.38187

Negative values indicate downregulation in BBP, while positive values indicate upregulation in BBP.

2. Quality control (QC) for PDAC study

The variability in the performance of the LC-MS system was evaluated by comparing the replicates of the Hela protein digest (Hela_QC) and the sample peptide pool (Pool_QC), respectively. The Hela_QC and Pool_QC replicates were analysed along with the samples of interest, with the Hela_QC replicates placed before, during at the end of the sample batches analysed, while the Pool_QC replicates were analysed at random with the samples of interest.

To assess the variability between the Hela_QC and Pool_QC replicates, the number of protein groups and peptides identified per replicate was compared. The Hela_QC replicates had a

similar number of protein groups and peptides that were identified, except for Hela_QC 4, which showed a possible error in sample injection (**Figure S1**). The Pool_QC replicates also showed that a similar number of protein groups and peptides were identified (**Figure S2**).

Additionally, Pearson correlations of the protein groups and peptides identified between the respective QC replicates were performed. A strong positive correlation of the protein groups ($\geq$ 0.942) and peptides ($\geq$ 0.843) identified, was observed between the Hela_QC replicates (**Figure S3** and **S4**, respectively), except for the Hela_QC 4 comparison with other replicates. Similarly, a strong positive correlation of the protein groups ($\geq$ 0.961) and peptides ($\geq$ 0.88) identified, was observed between the Pool_QC replicates (**Figure S5** and **S6**, respectively).

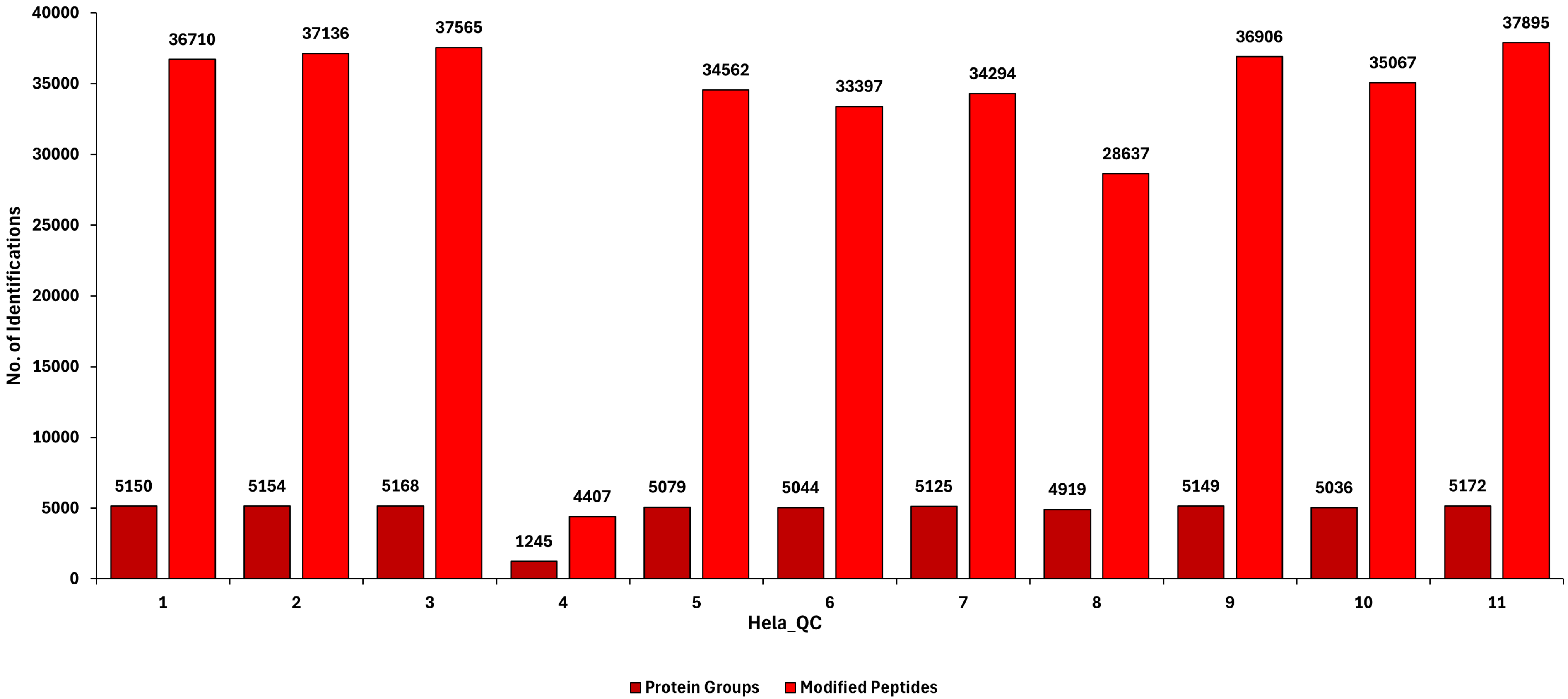

Figure S1: Bar graphs indicating the number of protein groups and modified peptides identified in each Hela_QC replicate. Protein groups are shown in dark red and the modified peptides in light red.

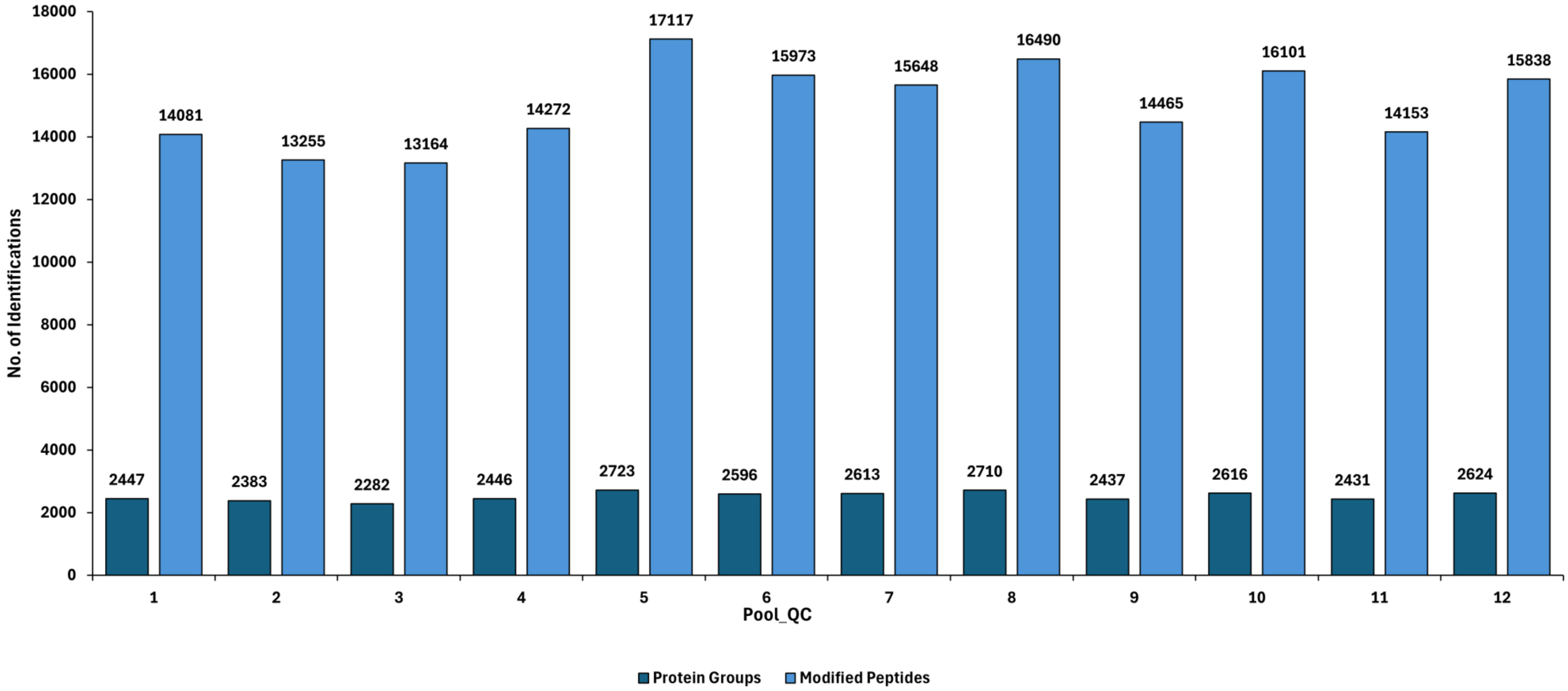

Figure S2: Bar graphs indicating the number of protein groups and modified peptides identified in each Pool_QC replicate. Protein groups in dark blue and modified peptides in light blue.

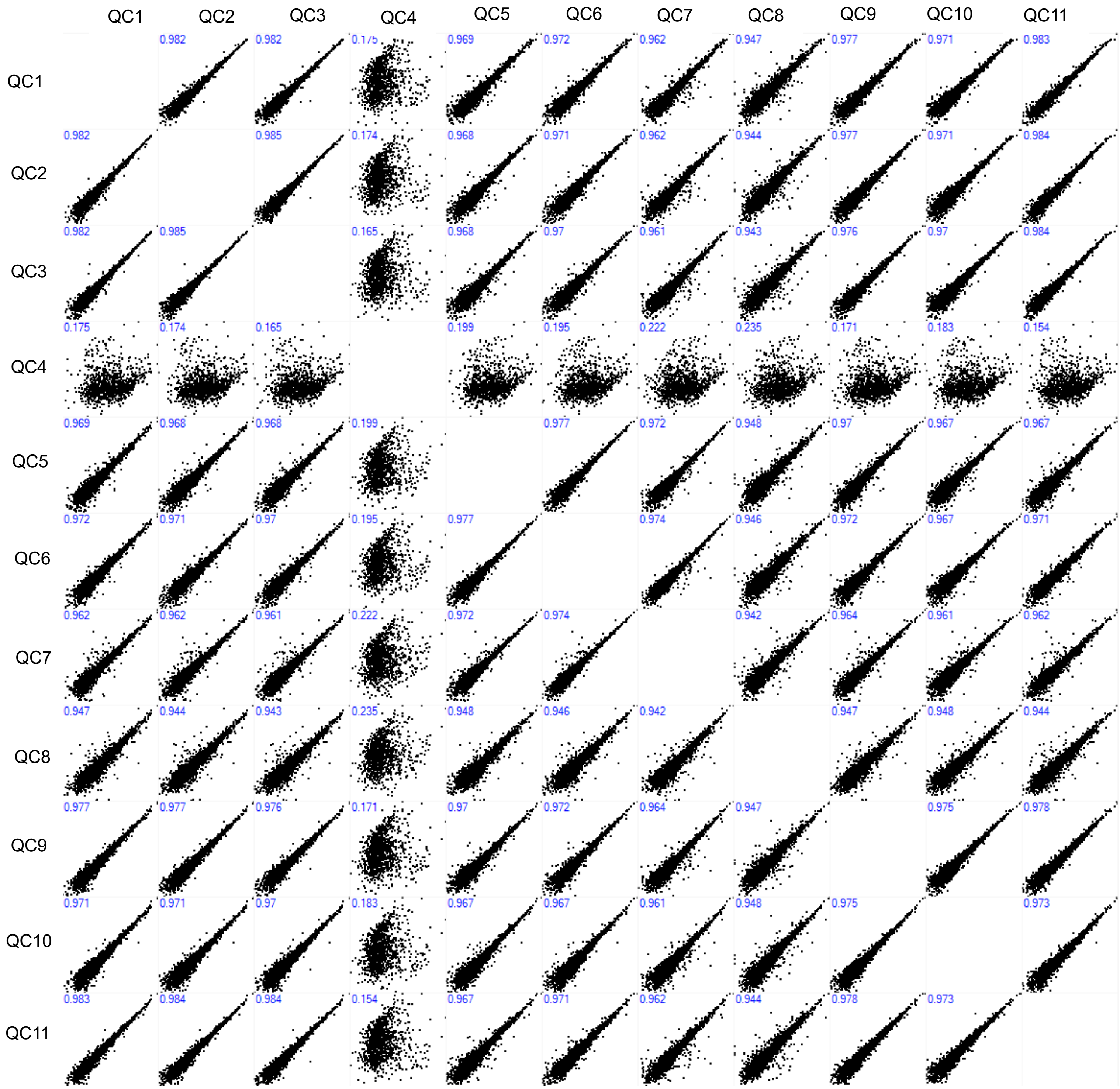

Figure S3: Multi-scatter plots showing Pearson correlation of identified protein groups between the Hela_QC replicates.

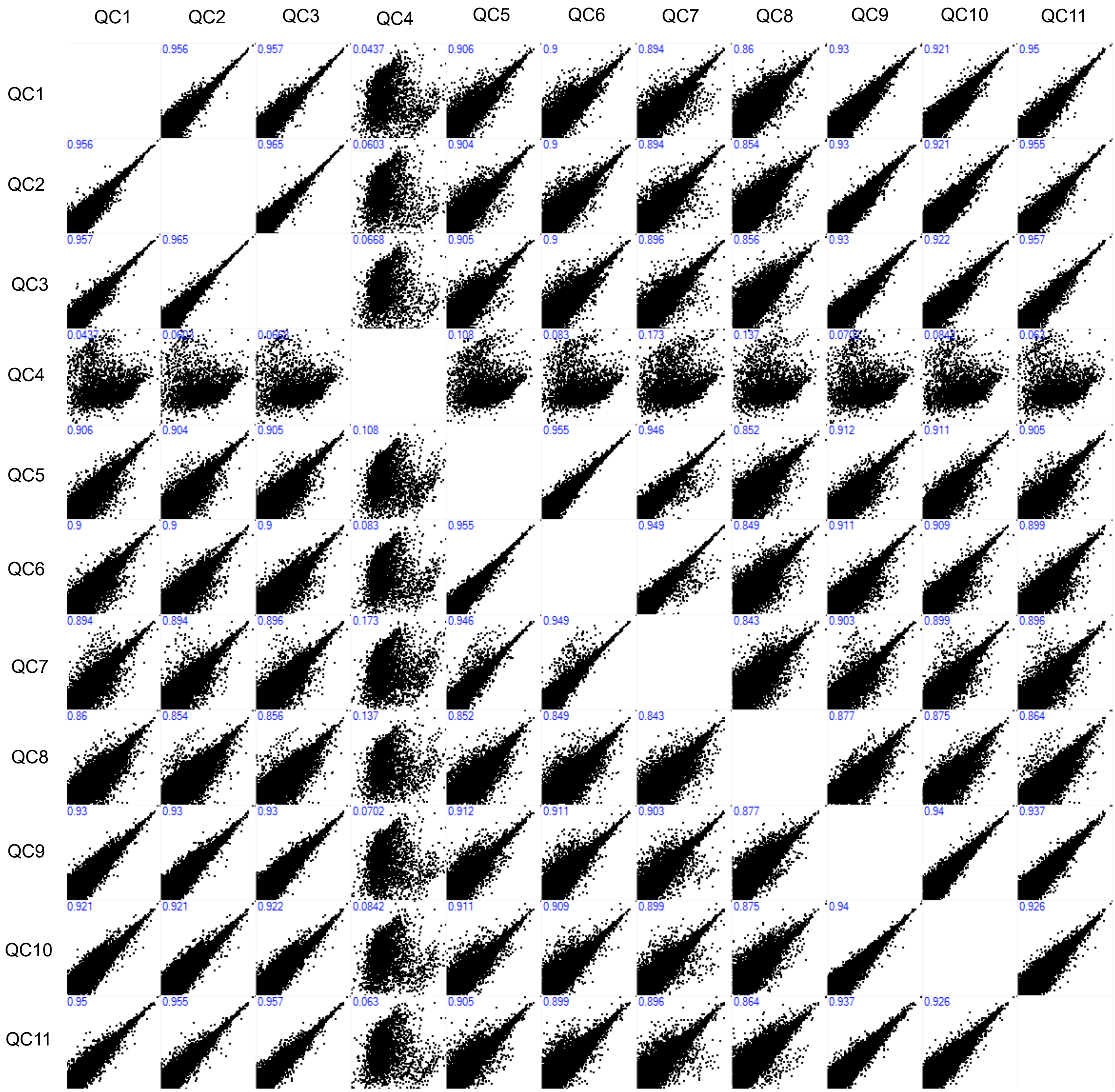

Figure S4: Multi-scatter plots showing Pearson correlation of identified peptides between the Hela_QC replicates.

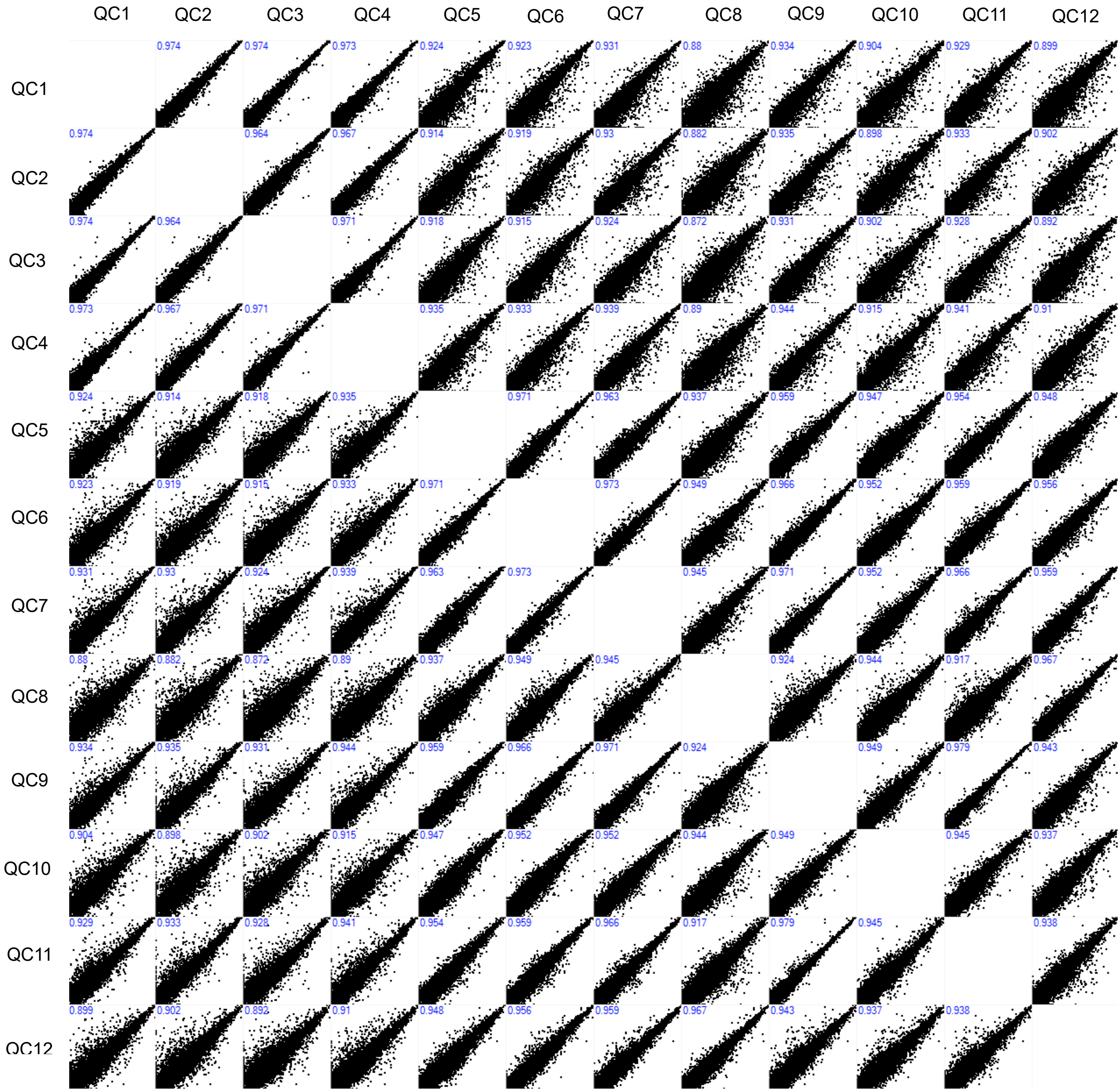

Figure S5: Multi-scatter plots showing Pearson correlation of identified protein groups between the Pool_QC replicates.

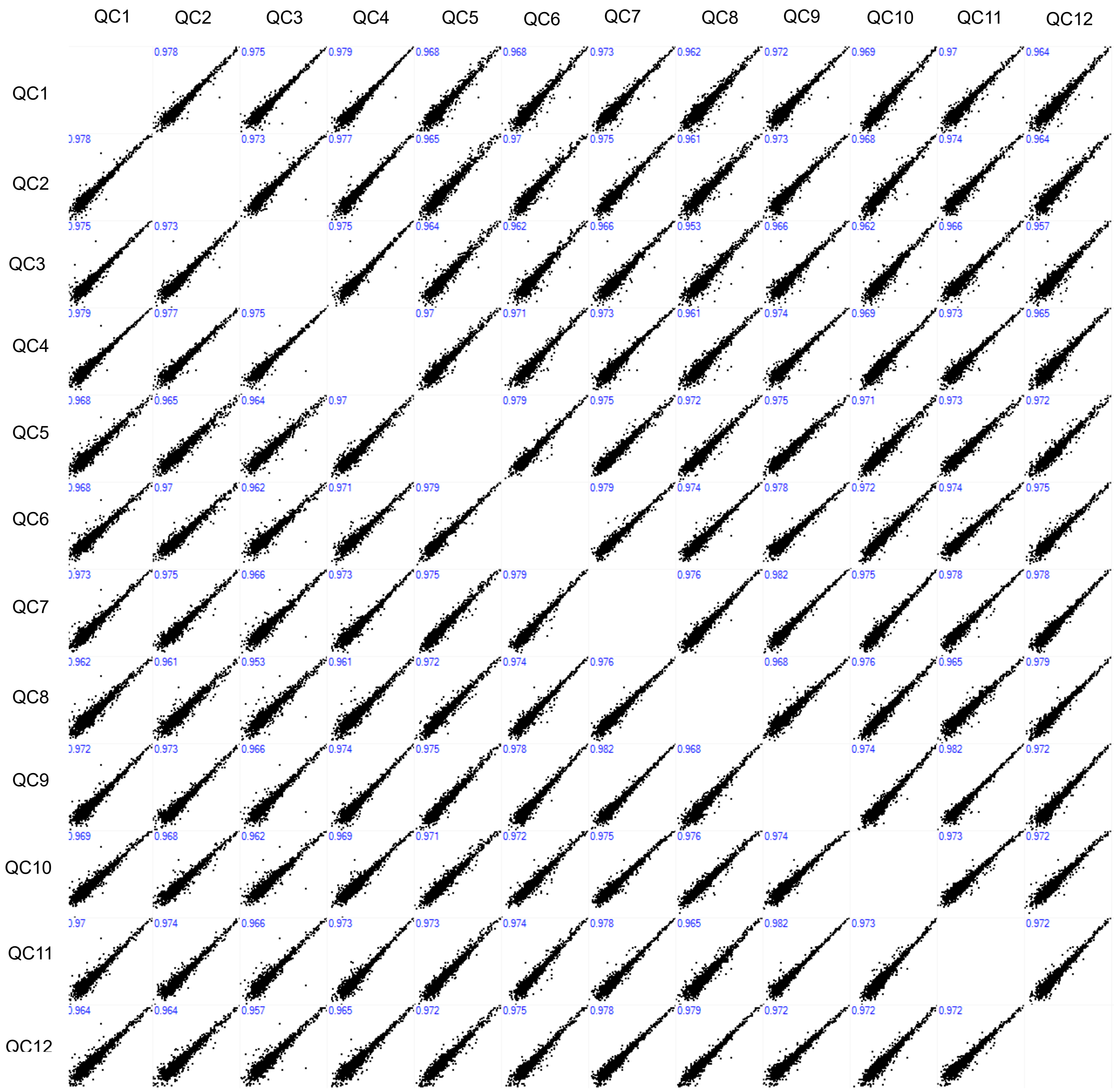

Figure S6: Multi-scatter plots showing Pearson correlation of identified peptides between the Pool_QC replicates.

In addition, the median percentage coefficient of variation (CV) shown below (**Figure S7**), illustrates the variation observed between the protein groups identified in the Hela_QC and Pool_QC replicates, respectively. The Hela_QC and Pool_QC replicates had a median CV of 19,6% and 23,5%, respectively, indicating that there is low technical variation between the replicates of both QC measurements.

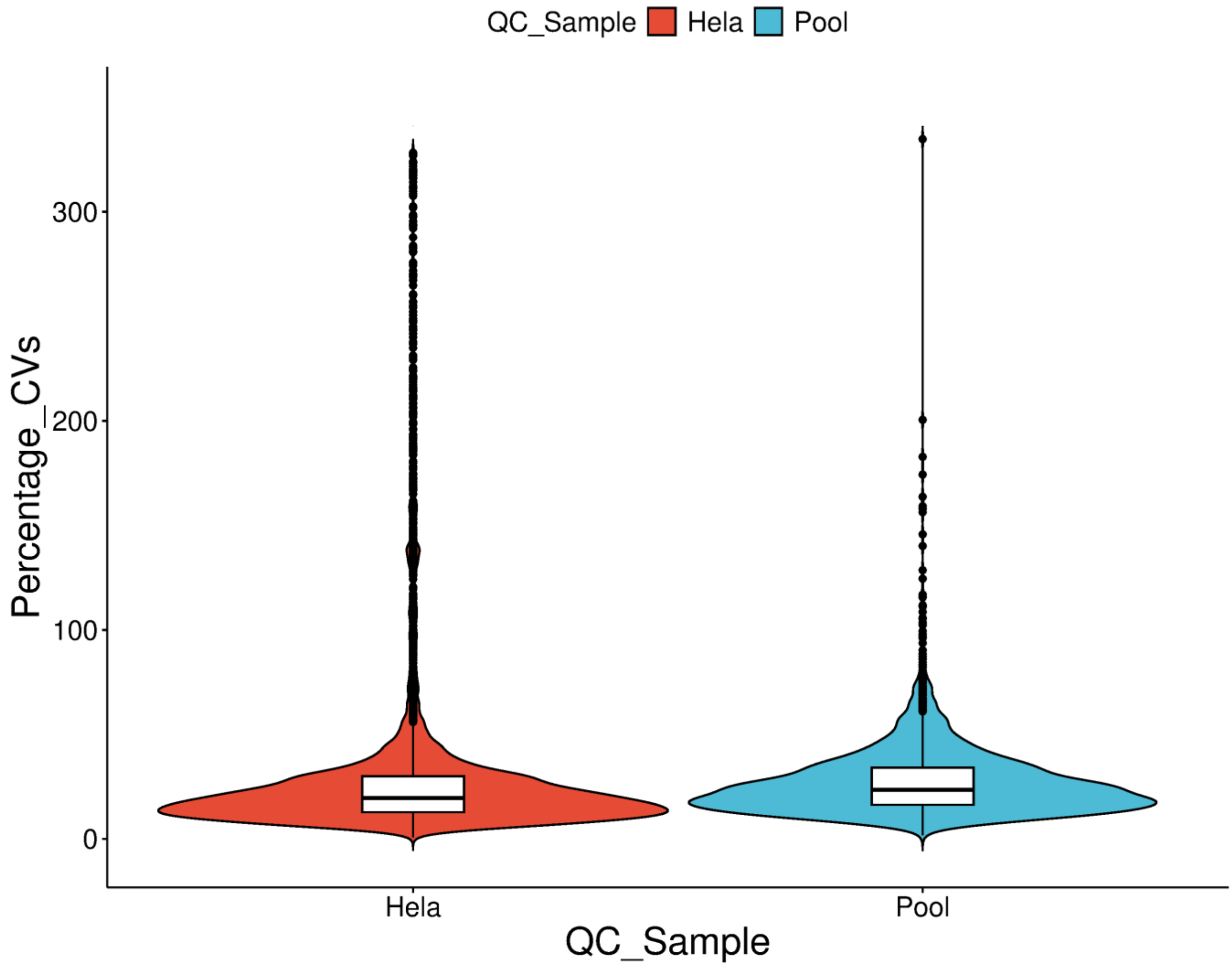

Figure S7: Variability between QC replicates. Violin plot indicating the median percentage coefficient of variation (% CV) between the Hela_QC (red) and Pool_QC (blue) replicates.

To further assess the quality and reliability of the experimental data, LC-derived peak parameters were tabulated below. Peak capacity is a common metric of separation power in gradient elution, and the typical peak capacity for peptide separation ranges from 100 to 400. The peak capacity for the Hela_QC replicates ranges between 122 - 144 and for the Pool_QC replicates ranges between 180 – 199, indicating that all the replicates are within the expected range for peptide separation (**Table S4**).

Furthermore, the decline in peak capacity results in the median full width half maximum (FWHM) of the peaks widening (indicated by an increase in the median FWHM), causing reduced peak resolution. Additionally, the average data points per peak for the Hela_QC and Pool_QC replicates are ~ 6 and 7, respectively (**Table S4**). The observed average data points per peak are sufficient for reproducible quantitation of complex samples.

Table S4: Table of LC derived peak parameters. The average data points per peak, peak capacity and the median FWHM in minutes is tabulated for each Hela_QC and Pool_QC replicate analysed.

	Average data points per peak	Peak Capacity	Median FWHM (minutes)
**Hela_QC 1**	6	123.62	0.054771
**Hela_QC 2**	6	127.73	0.053007
**Hela_QC 3**	6	126.65	0.053461
**Hela_QC 4**	6	131.32	0.051489
**Hela_QC 5**	6	121.99	0.055503
**Hela_QC 6**	6	122.82	0.055053
**Hela_QC 7**	6	125.66	0.053881
**Hela_QC 8**	5	131.45	0.051436
**Hela_QC 9**	5	136.96	0.049437
**Hela_QC 10**	5	143.15	0.047301
**Hela_QC 11**	6	125.52	0.053944
**Average data points per peak**	5.73		
**Pool_QC 1**	7	185.63	0.066651
**Pool_QC 2**	7	198.52	0.062323
**Pool_QC 3**	7	182.44	0.067816
**Pool_QC 4**	7	191.56	0.064588
**Pool_QC 5**	7	183.45	0.067443
**Pool_QC 6**	7	186.01	0.066514
**Pool_QC 7**	7	183.58	0.067394
**Pool_QC 8**	7	190.08	0.065091
**Pool_QC 9**	7	186.49	0.066343
**Pool_QC 10**	7	187.3	0.066056
**Pool_QC 11**	7	194.69	0.063549
**Pool_QC 12**	8	180.69	0.068475
**Average data points per peak**	7.08		

The Hela_QC and Pool_QC replicates served as system-suitability and sample-specific system-suitability measures, respectively, and were incorporated into the PDAC study to assess the LC-MS/MS system's performance variability. The variability in LC-MS/MS performance was evaluated at a peptide and protein level; **Figures S1 - 6** as well as **Table S4** showed that Hela_QC and Pool_QC replicates had low technical variation and that the peak related parameters performed adequately for reproducible quantitation of the complex samples of interest, suggesting that the data from the proteome profiling of this PDAC cohort was reliably and reproducibly acquired.

3. **List of dysregulated proteins post gender and age adjustment**

The study cohort comprised 81 patients diagnosed with PDAC and 97 control participants, including 62 individuals with benign biliary pathologies (BBP) and 35 healthy controls (HC). The BBP group consisted predominantly of females (85.5%), while PDAC cases were more commonly observed in males (67.1%; $p < 0.01$). The HC group showed a comparatively balanced sex distribution, with 42.9% females and 57.1% males (Figure S8). After adjusting for age and gender, most proteins retained their original significance across all comparisons. Specifically, in the comparisons, 279 proteins remained significant in the PDAC vs HC group, 351 in the PDAC vs BBP group, and 144 in the BBP vs HC group. Additionally, a subset of proteins either gained or lost significance after adjustment. For PDAC vs HC, 76 proteins gained significance and 104 lost significance. In the PDAC vs BBP comparison, 43 proteins gained significance and 95 lost significance. For BBP vs HC, 13 proteins gained significance and 60 lost significance (Table S5). Tables S6-8 indicate the list of differential proteins post age and gender adjustment across the PDAC vs HC, PDAC vs BBP, and BBP vs HC comparisons. These tables include the corresponding log-fold change values and statistical significance measures. Positive log-fold-change values indicate proteins that are upregulated, while negative values indicate proteins that are downregulated.

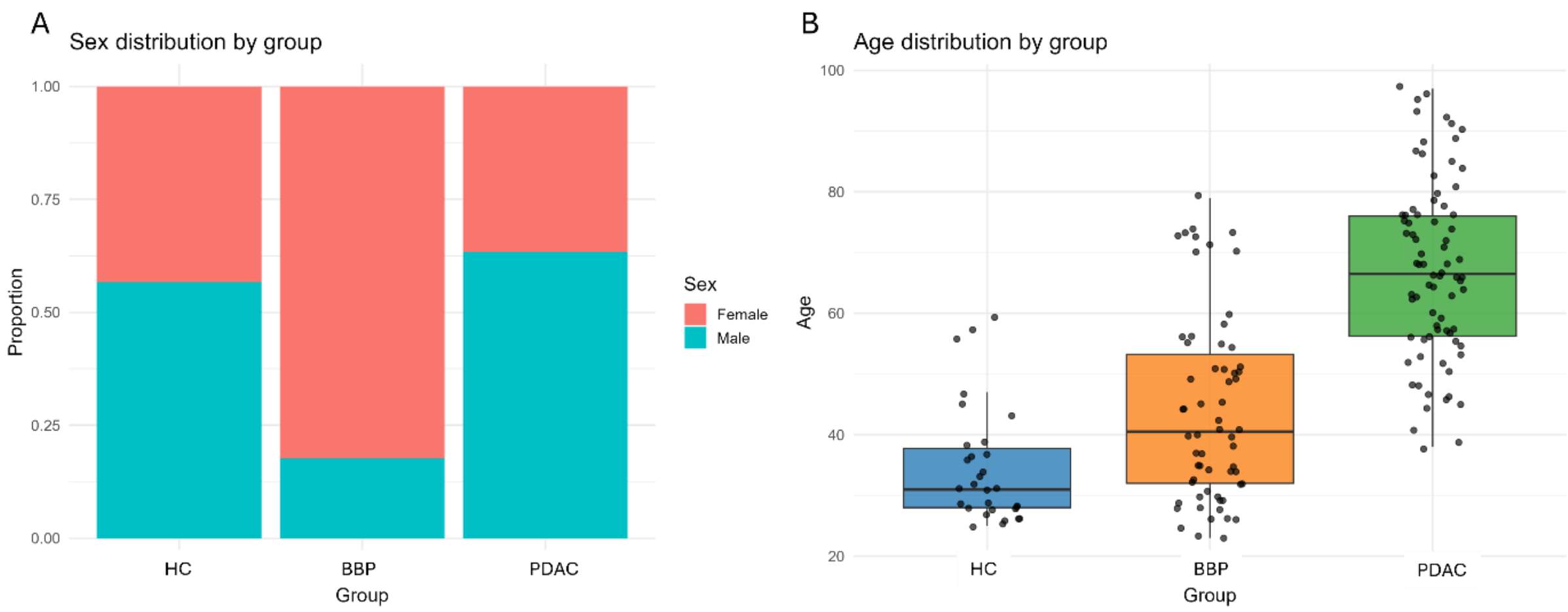

Figure S8: Bar and box blots indicating gender and age distribution for the PDAC, BBP and HC patient cohort. (A) Shows gender distribution and (B) shows age distribution.

Table S5: Summary of differential protein significance changes following age and gender adjustment across the PDAC vs HC, PDAC vs BBP, and BBP vs HC comparisons.

contrast	status	n
BBP_vs_HC	Gained after adjustment	13
BBP_vs_HC	Lost after adjustment	60
BBP_vs_HC	Not significant in either	1432
BBP_vs_HC	Retained after adjustment	144
PDAC_vs_BBP	Gained after adjustment	43
PDAC_vs_BBP	Lost after adjustment	95
PDAC_vs_BBP	Not significant in either	1160
PDAC_vs_BBP	Retained after adjustment	351
PDAC_vs_HC	Gained after adjustment	76
PDAC_vs_HC	Lost after adjustment	104
PDAC_vs_HC	Not significant in either	1190
PDAC_vs_HC	Retained after adjustment	279

Table S6: List of dysregulated proteins in BBP compared with HC post gender and age adjustment.

PG.Genes	logFC	AveExpr	t	P.Value	adj.P.Val	B	significant_FDR_0.05	significant_FDR_and_logFC
C1QTNF3	-1.6	4.798463	-6.77214	1.93E-10	6.44E-08	13.43688	TRUE	TRUE
APOA4	-1.5	9.562325	-5.1525	6.99E-07	4.68E-05	5.620727	TRUE	TRUE
CNDP1	-1.4	5.096258	-7.53003	2.74E-12	2.75E-09	17.5173	TRUE	TRUE
ENO3	-1.2	6.266695	-4.0041	9.24E-05	0.001934	1.030378	TRUE	TRUE
COG7	-1.2	9.259123	-4.05896	7.47E-05	0.001732	1.228726	TRUE	TRUE
PON3	-1.1	5.103396	-3.70384	0.000286	0.004221	-0.01521	TRUE	TRUE
IGFALS	-1.0	7.308555	-4.49847	1.26E-05	0.000548	2.896126	TRUE	TRUE
APOA2	-1.0	5.58073	-4.18068	4.62E-05	0.001288	1.676679	TRUE	TRUE

CETP	-1.0	5.150869	-3.82114	0.000185	0.003003	0.385139	TRUE	TRUE
LPA	-1.0	8.598404	-2.04572	0.042307	0.162982	-4.4676	FALSE	FALSE
MTTP	-1.0	3.761397	-3.98428	9.98E-05	0.002045	0.959291	TRUE	TRUE
PLXDC1	-1.0	4.071255	-5.78567	3.34E-08	6.71E-06	8.50991	TRUE	TRUE
GPNMB	1.0	6.496311	3.187516	0.001705	0.016617	-1.64903	TRUE	TRUE
PIGR	1.0	4.682228	4.222714	3.9E-05	0.001172	1.833851	TRUE	TRUE
TRAP1	1.0	8.640996	5.694683	5.24E-08	7.52E-06	8.080617	TRUE	TRUE
ANPEP	1.1	5.464543	4.928283	1.94E-06	0.000115	4.655424	TRUE	TRUE
HP	1.2	7.104812	4.383669	2.03E-05	0.000726	2.447403	TRUE	TRUE
H4C1	1.2	8.40442	3.514037	0.000564	0.006991	-0.64044	TRUE	TRUE
PRSS2	1.3	5.595533	3.127399	0.002071	0.018905	-1.82527	TRUE	TRUE
SAA1	1.4	6.531115	3.53067	0.000532	0.006761	-0.58678	TRUE	TRUE
MBL2	1.5	7.320854	3.154432	0.001898	0.018151	-1.74639	TRUE	TRUE
OIT3	1.5	6.335819	6.587075	5.25E-10	1.32E-07	12.47694	TRUE	TRUE
CCDC47	2.1	6.632109	5.696774	5.19E-08	7.52E-06	8.090432	TRUE	TRUE
CDHR2	3.4	7.841879	7.350742	7.64E-12	3.83E-09	16.53173	TRUE	TRUE

Table S7: List of dysregulated proteins in PDAC compared with HC post gender and age adjustment.

PG.Genes	logFC	AveExpr	t	P.Value	adj.P.Val	B	significant_FDR_0.05	significant_FDR_and_logFC
C1QTNF3	-2.0	4.798463	-7.00271	5.41E-11	4.94E-09	14.63492	TRUE	TRUE
APOA4	-1.8	9.562325	-5.21565	5.21E-07	1.09E-05	5.80274	TRUE	TRUE
TLN1	-1.8	8.957165	-3.66126	0.000334	0.002162	-0.30211	TRUE	TRUE
PFN1	-1.7	8.208926	-4.58047	8.87E-06	0.00011	3.104764	TRUE	TRUE
MADCAM1	-1.6	8.028279	-5.80254	3.07E-08	9.95E-07	8.51725	TRUE	TRUE
SLC4A1	-1.6	6.504238	-4.0273	8.45E-05	0.000695	0.979742	TRUE	TRUE
TUBA4A	-1.6	9.615406	-3.57656	0.000452	0.002752	-0.58395	TRUE	TRUE
GPLD1	-1.6	8.535957	-5.85963	2.31E-08	7.99E-07	8.791785	TRUE	TRUE
ACTN2	-1.5	8.763094	-3.54737	0.000502	0.002962	-0.67977	TRUE	TRUE
ZYX	-1.5	7.025836	-4.15652	5.09E-05	0.000473	1.456374	TRUE	TRUE
TUBB1	-1.5	9.192946	-3.02317	0.002884	0.012645	-2.28302	TRUE	TRUE
LPA	-1.4	8.598404	-2.58419	0.010591	0.033756	-3.44787	TRUE	TRUE
ILK	-1.4	7.755645	-3.70506	0.000285	0.001918	-0.15415	TRUE	TRUE
AHSG	-1.4	7.953429	-5.4723	1.55E-07	4.2E-06	6.965059	TRUE	TRUE
FERMT3	-1.4	7.652493	-4.10049	6.35E-05	0.000554	1.248191	TRUE	TRUE

PDLIM1	-1.4	7.884023	-3.24815	0.001397	0.006908	-1.62255	TRUE	TRUE
PON1	-1.4	7.341541	-4.66108	6.28E-06	8.3E-05	3.432413	TRUE	TRUE
PARVB	-1.3	7.08794	-3.66392	0.000331	0.002155	-0.29316	TRUE	TRUE
MYL6	-1.3	6.806574	-3.13308	0.002034	0.009497	-1.96563	TRUE	TRUE
SELENOP	-1.3	8.864454	-7.07418	3.63E-11	3.65E-09	15.02019	TRUE	TRUE
TAGLN2	-1.3	5.849566	-4.0119	8.97E-05	0.000725	0.923774	TRUE	TRUE
IGFALS	-1.3	7.308555	-4.84643	2.79E-06	4.01E-05	4.202367	TRUE	TRUE
FLNA	-1.3	10.46272	-2.38113	0.018352	0.051444	-3.92971	FALSE	FALSE
TPM4	-1.3	8.534102	-2.68659	0.007926	0.027348	-3.19107	TRUE	TRUE
GP5	-1.3	7.116923	-3.80827	0.000194	0.001385	0.200363	TRUE	TRUE
CAVIN2	-1.3	6.901364	-3.39507	0.000852	0.004598	-1.16868	TRUE	TRUE
MYH9	-1.3	8.986699	-2.45723	0.014993	0.043886	-3.75343	TRUE	TRUE
ACTN1	-1.3	8.288351	-3.13634	0.002013	0.009442	-1.95606	TRUE	TRUE
MTTP	-1.3	3.761397	-4.35576	2.27E-05	0.000251	2.215181	TRUE	TRUE
PON3	-1.3	5.103396	-3.69828	0.000292	0.001953	-0.17717	TRUE	TRUE
ATP2A3	-1.3	6.888712	-3.34233	0.001019	0.005386	-1.33363	TRUE	TRUE
PKM	-1.2	6.798989	-4.52771	1.11E-05	0.000133	2.892759	TRUE	TRUE

GPX1	-1.2	5.640162	-4.03179	8.3E-05	0.000689	0.9961	TRUE	TRUE
APOL1	-1.2	9.57954	-5.02241	1.27E-06	2.23E-05	4.954327	TRUE	TRUE
APOA2	-1.2	5.58073	-4.19239	4.41E-05	0.000443	1.59087	TRUE	TRUE
TMSB4X	-1.2	6.183083	-3.43943	0.000731	0.004057	-1.0282	TRUE	TRUE
CNDP1	-1.2	5.096258	-5.29775	3.55E-07	7.92E-06	6.170241	TRUE	TRUE
ITGB3	-1.2	8.570945	-2.6965	0.007704	0.027036	-3.16572	TRUE	TRUE
PLEK	-1.2	4.284559	-3.87219	0.000153	0.001121	0.424068	TRUE	TRUE
RSU1	-1.2	7.105889	-3.73553	0.000255	0.00175	-0.05034	TRUE	TRUE
WDR1	-1.2	6.897957	-3.80129	0.0002	0.001411	0.17613	TRUE	TRUE
LDHB	-1.2	5.590964	-3.97099	0.000105	0.00083	0.7759	TRUE	TRUE
RAC1	-1.2	5.855873	-5.35112	2.76E-07	6.44E-06	6.411302	TRUE	TRUE
VCL	-1.2	6.455689	-3.4982	0.000596	0.003401	-0.83965	TRUE	TRUE
ABRACL	-1.2	4.204393	-4.30097	2.84E-05	0.000307	2.003636	TRUE	TRUE
APEH	-1.1	5.932669	-3.52218	0.000548	0.003163	-0.76193	TRUE	TRUE
ACTG1	-1.1	7.555101	-2.74032	0.006786	0.024418	-3.05265	TRUE	TRUE
COG7	-1.1	9.259123	-3.33251	0.001054	0.005524	-1.3641	TRUE	TRUE
ITGA2B	-1.1	10.31619	-2.46497	0.014684	0.043362	-3.73522	TRUE	TRUE

CORO1C	-1.1	7.062162	-2.62018	0.009574	0.030909	-3.35867	TRUE	TRUE
CAP1	-1.1	6.300797	-3.56299	0.000475	0.002853	-0.62857	TRUE	TRUE
GP1BB	-1.1	9.01909	-2.45133	0.015233	0.044201	-3.76729	TRUE	TRUE
MYL9	-1.1	7.014532	-2.67702	0.008147	0.027916	-3.21546	TRUE	TRUE
CD9	-1.1	6.75744	-3.27035	0.001298	0.00655	-1.55511	TRUE	TRUE
PRDX6	-1.1	6.287114	-4.94156	1.83E-06	2.84E-05	4.606382	TRUE	TRUE
CFL1	-1.0	4.896763	-4.18575	4.53E-05	0.00045	1.565886	TRUE	TRUE
LIMS1	-1.0	6.862649	-3.24842	0.001396	0.006908	-1.62173	TRUE	TRUE
VASN	-1.0	5.384313	-4.85498	2.69E-06	3.91E-05	4.238446	TRUE	TRUE
STXBP2	-1.0	5.468036	-3.89007	0.000143	0.001072	0.48717	TRUE	TRUE
VCP	-1.0	8.90907	-3.48612	0.000622	0.003504	-0.87864	TRUE	TRUE
ANK1	-1.0	6.498953	-4.30703	2.77E-05	0.000303	2.026958	TRUE	TRUE
CCDC126	-1.0	3.392085	-3.97566	0.000103	0.000822	0.792707	TRUE	TRUE
STIM1	-1.0	5.553111	-2.88167	0.00446	0.017983	-2.67658	TRUE	TRUE
MGLL	-1.0	4.374472	-3.79388	0.000205	0.00144	0.15046	TRUE	TRUE
EHD3	-1.0	5.043312	-3.44697	0.000713	0.003975	-1.00417	TRUE	TRUE
GNAQ	-1.0	7.418997	-3.52974	0.000534	0.003116	-0.73734	TRUE	TRUE

CYB5R3	-1.0	7.181758	-2.48563	0.013887	0.041496	-3.68633	TRUE	TRUE
NCAM1	-1.0	9.267737	-5.53236	1.16E-07	3.23E-06	7.242685	TRUE	TRUE
MAPRE2	-1.0	6.109528	-3.08626	0.002363	0.010783	-2.10205	TRUE	TRUE
MDH2	-1.0	5.887924	-2.66375	0.008462	0.028509	-3.24915	TRUE	TRUE
PLXDC1	-1.0	4.071255	-4.83543	2.93E-06	4.15E-05	4.156054	TRUE	TRUE
PSMB10	1.0	4.888288	4.46605	1.44E-05	0.000168	2.647384	TRUE	TRUE
TRAP1	1.0	8.640996	4.427411	1.69E-05	0.000193	2.494986	TRUE	TRUE
ADIPOQ	1.0	4.547776	4.427127	1.69E-05	0.000193	2.49387	TRUE	TRUE
TFPI	1.0	5.327868	5.278226	3.89E-07	8.49E-06	6.082477	TRUE	TRUE
IGKV1-27	1.0	6.166196	2.911161	0.004078	0.016711	-2.59597	TRUE	TRUE
CLEC11A	1.0	7.718111	4.239048	3.66E-05	0.000376	1.7672	TRUE	TRUE
ADAMTSL2	1.0	5.447708	5.942525	1.52E-08	5.65E-07	9.19359	TRUE	TRUE
C8G	1.0	5.726652	5.227468	4.93E-07	1.05E-05	5.855402	TRUE	TRUE
CDH6	1.0	5.674345	4.709107	5.1E-06	7.02E-05	3.629716	TRUE	TRUE
C1QB	1.0	5.884644	6.321399	2.15E-09	1.14E-07	11.07585	TRUE	TRUE
LDLR	1.1	6.389474	4.140753	5.41E-05	0.000491	1.397559	TRUE	TRUE
FBLN5	1.1	9.905659	4.978401	1.55E-06	2.59E-05	4.764406	TRUE	TRUE
DCN	1.1	5.064752	4.116683	5.95E-05	0.000529	1.30812	TRUE	TRUE
LTBP4	1.1	4.616053	4.985251	1.5E-06	2.55E-05	4.793888	TRUE	TRUE
AXL	1.1	6.834501	4.681007	5.76E-06	7.82E-05	3.514089	TRUE	TRUE
PTMA	1.1	7.584117	3.06721	0.00251	0.011353	-2.15705	TRUE	TRUE
CAMP	1.1	4.96452	4.603745	8.03E-06	0.000103	3.19892	TRUE	TRUE

PSMB9	1.1	5.631854	4.955391	1.72E-06	2.83E-05	4.6656	TRUE	TRUE
A1BG	1.1	6.42221	5.852255	2.39E-08	8.01E-07	8.756237	TRUE	TRUE
ORM1	1.1	8.612303	4.593191	8.4E-06	0.000107	3.156181	TRUE	TRUE
LYVE1	1.1	4.939223	5.761053	3.78E-08	1.18E-06	8.318878	TRUE	TRUE
EMILIN2	1.1	5.773272	4.949719	1.76E-06	2.83E-05	4.641296	TRUE	TRUE
MXRA5	1.1	4.715037	5.093951	9.15E-07	1.73E-05	5.2657	TRUE	TRUE
FCGBP	1.1	5.189067	4.558929	9.72E-06	0.000118	3.017972	TRUE	TRUE
S100A9	1.2	5.71744	4.04136	8E-05	0.000677	1.030995	TRUE	TRUE
PRR4	1.2	6.485485	2.944214	0.003685	0.015417	-2.50473	TRUE	TRUE
ITIH3	1.2	13.20688	7.960132	2.23E-13	2.23E-10	19.95912	TRUE	TRUE
PRAP1	1.2	4.539093	4.17355	4.75E-05	0.000463	1.520108	TRUE	TRUE
ILF3	1.2	4.953565	5.447041	1.75E-07	4.5E-06	6.8489	TRUE	TRUE
C1QC	1.2	6.407636	7.231023	1.51E-11	2.16E-09	15.87295	TRUE	TRUE
HNRNPU	1.2	5.389558	4.238105	3.67E-05	0.000376	1.763618	TRUE	TRUE
CFI	1.2	5.303596	7.727872	8.69E-13	3.72E-10	18.63693	TRUE	TRUE
ICAM1	1.3	5.011326	6.160065	4.99E-09	2.18E-07	10.26533	TRUE	TRUE
TGFBI	1.3	8.566562	5.108957	8.54E-07	1.65E-05	5.331423	TRUE	TRUE
SPP1	1.3	6.901615	5.010035	1.34E-06	2.32E-05	4.900799	TRUE	TRUE
S100A12	1.3	3.116113	4.885557	2.35E-06	3.49E-05	4.367828	TRUE	TRUE
AEBP1	1.3	6.769765	5.399637	2.19E-07	5.24E-06	6.631965	TRUE	TRUE
VCAN	1.3	6.879798	4.940168	1.84E-06	2.84E-05	4.600419	TRUE	TRUE
SRPX	1.3	5.370917	5.303642	3.45E-07	7.88E-06	6.196768	TRUE	TRUE
ITIH4	1.3	7.962594	6.210206	3.85E-09	1.76E-07	10.51582	TRUE	TRUE
EFEMP2	1.3	5.482197	6.051927	8.71E-09	3.64E-07	9.729477	TRUE	TRUE
HP	1.3	7.104812	4.293431	2.93E-05	0.00031	1.974719	TRUE	TRUE

CSE1L	1.4	5.289416	3.579279	0.000448	0.002752	-0.575	TRUE	TRUE
RPLP1	1.4	6.736593	4.664857	6.18E-06	8.27E-05	3.447874	TRUE	TRUE
P4HA1	1.4	4.326596	6.372324	1.65E-09	9.73E-08	11.33437	TRUE	TRUE
IGFBP1	1.4	6.479194	4.496336	1.27E-05	0.00015	2.767568	TRUE	TRUE
MFAP4	1.4	4.732411	4.040633	8.02E-05	0.000677	1.028342	TRUE	TRUE
ILF2	1.4	3.714696	5.709851	4.86E-08	1.48E-06	8.075357	TRUE	TRUE
MIA3	1.5	5.867774	5.86035	2.3E-08	7.99E-07	8.795275	TRUE	TRUE
RPLP2	1.5	7.315711	5.424042	1.95E-07	4.9E-06	6.743483	TRUE	TRUE
LTBP2	1.5	5.243999	6.407704	1.37E-09	8.57E-08	11.51473	TRUE	TRUE
GOLM1	1.5	8.955817	6.865701	1.15E-10	8.91E-09	13.90246	TRUE	TRUE
IGLL1	1.5	6.127657	3.757609	0.000235	0.001625	0.025322	TRUE	TRUE
VWF	1.6	8.998039	5.616349	7.7E-08	2.21E-06	7.634468	TRUE	TRUE
RPLP0	1.6	4.587538	5.203374	5.52E-07	1.11E-05	5.74817	TRUE	TRUE
THBS2	1.6	7.367611	5.987312	1.21E-08	4.86E-07	9.412204	TRUE	TRUE
LRG1	1.7	3.855639	7.319872	9.1E-12	1.52E-09	16.36038	TRUE	TRUE
ANPEP	1.7	5.464543	6.545219	6.57E-10	4.71E-08	12.22146	TRUE	TRUE
PTX3	1.8	7.557785	5.463832	1.61E-07	4.26E-06	6.92606	TRUE	TRUE
FBLN2	1.8	5.788112	7.11454	2.9E-11	3.23E-09	15.23866	TRUE	TRUE
OIT3	1.8	6.335819	6.502545	8.25E-10	5.52E-08	12.00118	TRUE	TRUE
PIGR	1.8	4.682228	6.349765	1.85E-09	1.03E-07	11.21969	TRUE	TRUE
CTSS	1.9	5.196432	7.633372	1.51E-12	3.78E-10	18.10426	TRUE	TRUE
DMBT1	2.0	5.471433	6.947148	7.36E-11	6.16E-09	14.33692	TRUE	TRUE
IGHG3	2.0	5.414292	5.079253	9.79E-07	1.82E-05	5.201466	TRUE	TRUE
GPNMB	2.1	6.496311	5.691431	5.33E-08	1.57E-06	7.988111	TRUE	TRUE
PRSS2	2.3	5.595533	4.629315	7.2E-06	9.39E-05	3.302777	TRUE	TRUE

SDC1	2.4	8.989741	7.415173	5.29E-12	1.06E-09	16.88662	TRUE	TRUE
H4C1	2.6	8.40442	6.253781	3.07E-09	1.47E-07	10.73455	TRUE	TRUE
SAA1	2.6	6.531115	5.399975	2.19E-07	5.24E-06	6.633506	TRUE	TRUE
CCDC47	3.1	6.632109	7.15484	2.31E-11	2.9E-09	15.45749	TRUE	TRUE
CDHR2	4.2	7.841879	7.685592	1.11E-12	3.72E-10	18.39822	TRUE	TRUE

Table S8: List of dysregulated proteins in PDAC compared with BBP post gender and age adjustment.

PG.Genes	logFC	AveExpr	t	P.Value	adj.P.Val	B	significant_FDR_0.05	significant_FDR_and_logFC
TUBB1	-1.6	9.192946	-4.01342	8.92E-05	0.001194	1.112031	TRUE	TRUE
TLN1	-1.6	8.957165	-3.98681	9.88E-05	0.001255	1.016821	TRUE	TRUE
FLNA	-1.6	10.46272	-3.47903	0.000638	0.004051	-0.69702	TRUE	TRUE
TPM4	-1.5	8.534102	-3.82489	0.000183	0.001853	0.448989	TRUE	TRUE
MYH9	-1.5	8.986699	-3.50443	0.000583	0.003853	-0.61604	TRUE	TRUE
ACTN2	-1.5	8.763094	-4.2543	3.44E-05	0.000712	1.997152	TRUE	TRUE
TUBA4A	-1.5	9.615406	-3.94462	0.000116	0.001389	0.86698	TRUE	TRUE
PDLIM1	-1.5	7.884023	-4.15353	5.15E-05	0.000807	1.621741	TRUE	TRUE
ACTN1	-1.4	8.288351	-4.19448	4.37E-05	0.000753	1.773434	TRUE	TRUE
SLC4A1	-1.4	6.504238	-4.18811	4.48E-05	0.000753	1.749755	TRUE	TRUE
ILK	-1.3	7.755645	-4.22954	3.8E-05	0.000728	1.904226	TRUE	TRUE

CAVIN2	-1.3	6.901364	-4.25169	3.47E-05	0.000712	1.987342	TRUE	TRUE
GP1BB	-1.3	9.01909	-3.45835	0.000685	0.004219	-0.76258	TRUE	TRUE
ACTB	-1.3	10.79936	-3.22052	0.00153	0.00728	-1.4921	TRUE	TRUE
PARVB	-1.3	7.08794	-4.2598	3.36E-05	0.000712	2.017834	TRUE	TRUE
ITGB3	-1.3	8.570945	-3.47058	0.000657	0.004102	-0.72387	TRUE	TRUE
ZYX	-1.3	7.025836	-4.24062	3.63E-05	0.000728	1.945756	TRUE	TRUE
ITGA2B	-1.3	10.31619	-3.314	0.001121	0.005759	-1.21079	TRUE	TRUE
MYL6	-1.2	6.806574	-3.55649	0.000486	0.003483	-0.44842	TRUE	TRUE
ACTG1	-1.2	7.555101	-3.56171	0.000477	0.003483	-0.4315	TRUE	TRUE
CORO1C	-1.2	7.062162	-3.4346	0.000744	0.004445	-0.83749	TRUE	TRUE
GPLD1	-1.2	8.535957	-5.28958	3.69E-07	4.12E-05	6.252022	TRUE	TRUE
MYL9	-1.2	7.014532	-3.47003	0.000658	0.004102	-0.72559	TRUE	TRUE
FERMT3	-1.2	7.652493	-4.18722	4.5E-05	0.000753	1.746428	TRUE	TRUE
GP5	-1.2	7.116923	-4.17316	4.76E-05	0.000759	1.694302	TRUE	TRUE
ATP2A3	-1.1	6.888712	-3.69442	0.000296	0.002558	0.005808	TRUE	TRUE
STOM	-1.1	7.726231	-3.31324	0.001124	0.005759	-1.21311	TRUE	TRUE
LIMS1	-1.1	6.862649	-4.2935	2.93E-05	0.000678	2.145148	TRUE	TRUE

MYH11	-1.1	7.560155	-2.78023	0.006037	0.020546	-2.72007	TRUE	TRUE
TUBA8	-1.1	6.945602	-3.47678	0.000643	0.004057	-0.70419	TRUE	TRUE
PFN1	-1.1	8.208926	-3.53681	0.000521	0.00363	-0.51205	TRUE	TRUE
CYB5R3	-1.1	7.181758	-3.32885	0.001067	0.005607	-1.16545	TRUE	TRUE
MAPRE2	-1.1	6.109528	-4.19808	4.31E-05	0.000753	1.786812	TRUE	TRUE
RSU1	-1.1	7.105889	-4.18076	4.62E-05	0.000759	1.722471	TRUE	TRUE
GPX1	-1.1	5.640162	-4.25583	3.42E-05	0.000712	2.002905	TRUE	TRUE
ARL6IP5	-1.1	6.088678	-3.26603	0.001316	0.00651	-1.35603	TRUE	TRUE
TPM3	-1.1	7.424836	-3.47925	0.000637	0.004051	-0.69633	TRUE	TRUE
RAB27B	-1.1	7.315621	-3.03288	0.002798	0.011464	-2.03519	TRUE	TRUE
TUBB4B	-1.1	10.0346	-3.40945	0.000811	0.004706	-0.9163	TRUE	TRUE
F11R	-1.1	7.537105	-3.58382	0.000441	0.003352	-0.35961	TRUE	TRUE
TUBB	-1.0	10.04399	-3.37676	0.000907	0.005058	-1.01798	TRUE	TRUE
ARPC4	-1.0	5.916292	-3.82754	0.000181	0.001853	0.458124	TRUE	TRUE
CLTC	-1.0	7.466698	-4.09432	6.5E-05	0.000933	1.404584	TRUE	TRUE
TPM2	-1.0	7.074969	-3.58554	0.000438	0.003352	-0.354	TRUE	TRUE
CAP1	-1.0	6.300797	-3.73575	0.000254	0.002343	0.144782	TRUE	TRUE

SPTB	-1.0	5.807131	-5.16842	6.49E-07	5.43E-05	5.718611	TRUE	TRUE
STIM1	-1.0	5.553111	-3.24773	0.001399	0.006787	-1.41094	TRUE	TRUE
NAP1L1	-1.0	6.789499	-3.78752	0.00021	0.002028	0.320738	TRUE	TRUE
ANK1	-1.0	6.498953	-4.78812	3.61E-06	0.000139	4.103551	TRUE	TRUE
CTSS	1.0	5.196432	4.78954	3.59E-06	0.000139	4.109391	TRUE	TRUE
P4HA1	1.0	4.326596	5.43278	1.87E-07	2.35E-05	6.893753	TRUE	TRUE
PRSS2	1.0	5.595533	2.419023	0.016604	0.043301	-3.60425	TRUE	TRUE
MXRA5	1.0	4.715037	5.558166	1.02E-07	1.61E-05	7.46545	TRUE	TRUE
VCAN	1.0	6.879798	4.647966	6.65E-06	0.00024	3.53181	TRUE	TRUE
CCDC47	1.0	6.632109	2.865719	0.00468	0.016962	-2.49436	TRUE	TRUE
VWF	1.0	8.998039	4.382385	2.04E-05	0.000531	2.484695	TRUE	TRUE
HSPG2	1.0	7.230206	5.150011	7.07E-07	5.44E-05	5.638362	TRUE	TRUE
TFRC	1.1	6.131638	4.487462	1.31E-05	0.000388	2.893217	TRUE	TRUE
SRPX	1.1	5.370917	5.169138	6.47E-07	5.43E-05	5.721762	TRUE	TRUE
EMILIN2	1.1	5.773272	5.664927	6.07E-08	1.38E-05	7.959266	TRUE	TRUE
ADIPOQ	1.1	4.547776	5.84263	2.51E-08	8.41E-06	8.795192	TRUE	TRUE
LRG1	1.1	3.855639	5.639089	6.89E-08	1.38E-05	7.839167	TRUE	TRUE
IGHG3	1.1	5.414292	3.327236	0.001073	0.005609	-1.17039	TRUE	TRUE
SPP1	1.1	6.901615	5.231714	4.84E-07	4.86E-05	5.99617	TRUE	TRUE
GPNMB	1.1	6.496311	3.638884	0.000362	0.002906	-0.17885	TRUE	TRUE
THBS2	1.1	7.367611	4.984504	1.51E-06	7.35E-05	4.926148	TRUE	TRUE

SAA1	1.2	6.531115	2.940416	0.003729	0.014421	-2.29208	TRUE	TRUE
DMBT1	1.2	5.471433	4.979899	1.54E-06	7.35E-05	4.906573	TRUE	TRUE
LTBP2	1.3	5.243999	6.35046	1.85E-09	9.28E-07	11.27519	TRUE	TRUE
IGLL1	1.3	6.127657	3.702562	0.000287	0.002529	0.033097	TRUE	TRUE
CSE1L	1.3	5.289416	4.108541	6.15E-05	0.000895	1.45652	TRUE	TRUE
PTX3	1.3	7.557785	4.863869	2.58E-06	0.000113	4.417892	TRUE	TRUE
FBLN2	1.3	5.788112	6.38022	1.58E-09	9.28E-07	11.4245	TRUE	TRUE
H4C1	1.4	8.40442	3.986725	9.89E-05	0.001255	1.016524	TRUE	TRUE
SDC1	1.5	8.989741	5.538676	1.12E-07	1.61E-05	7.375993	TRUE	TRUE

4. PCA projection

The PC1 vs PC2 projection primarily captured variance that did not spatially resolve the biological groups, whereas the projection presented in the main manuscript provided clearer biological separation between cohorts.

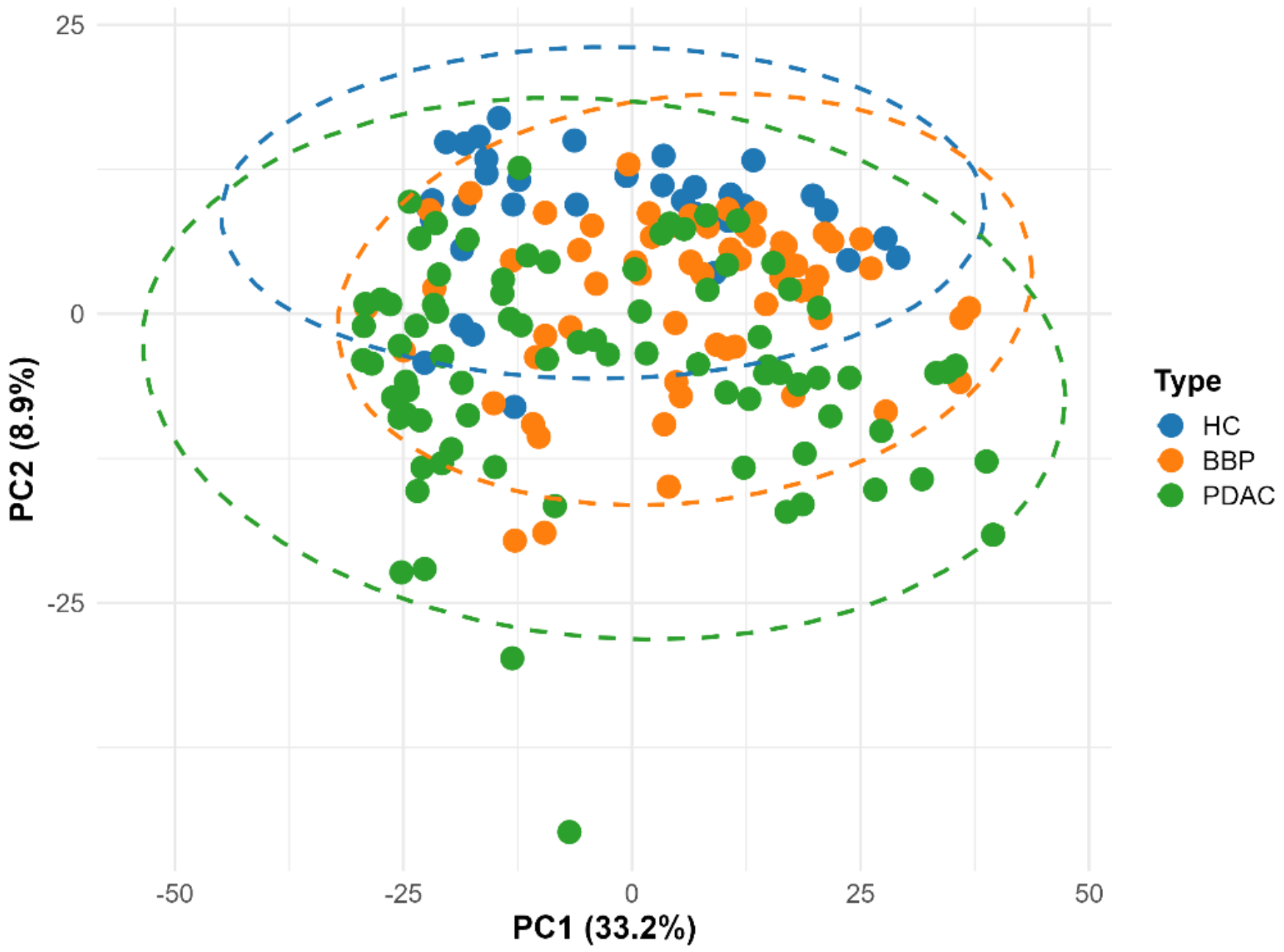

Figure S9: PCA of HC, BBP and PDAC protein profiles. The plot reveals clustering patterns in PC1 and PC2.
